# Comprehensive identification of risk factors for recurrence after percutaneous endoscopic lumbar discectomy: a systematic review and meta-analysis

**DOI:** 10.3389/fsurg.2026.1733796

**Published:** 2026-04-07

**Authors:** Abuduwupuer Haibier, Jia Wang, Wei Liu, Guanghui Wang

**Affiliations:** 1 Xinjiang Medical University, Urumqi, Xinjiang Uygur Autonomous Region, China; 2 Huashan Hospital Fudan University, Shanghai, China; 3 Shenzhen Second People’s Hospital, Shenzhen, Guangdong, China

**Keywords:** lumbar disc herniation, meta-analysis, percutaneous endoscopic lumbar discectomy, recurrence, risk factors

## Abstract

**Objective:**

This meta-analysis aimed to identify risk factors associated with postoperative recurrence following Percutaneous Endoscopic Lumbar Discectomy (PELD) for Lumbar Disc Herniation (LDH).

**Methods:**

We systematically searched PubMed, the Cochrane Library, EMbase, CNKI, WanFang, and VIP databases for case-control and cohort studies investigating risk factors for recurrence after PELD, from their inception until August 30, 2025. Two reviewers independently extracted data and assessed the methodological quality of the included studies using the Newcastle-Ottawa Scale (NOS). Meta-analysis was performed using Stata 12.0 software to calculate the pooled odds ratios (OR) and 95% confidence intervals (CI) for each potential factor.

**Results:**

A total of 39 case-control studies, involving 14,454 patients, were included. The overall postoperative recurrence rate was 11.0% (95% CI: 9.1%–13.0%). Factors significantly associated with an increased risk of recurrence included: Modic changes (OR = 1.74, 95% CI: 1.25–2.23), particularly type II Modic changes (OR = 1.87, 95% CI: 1.02–2.72); diabetes mellitus (OR = 2.34, 95% CI: 1.52–3.59); smoking (OR = 2.02, 95% CI: 1.27–3.21); intraoperative annulus fibrosus rupture (OR = 2.40, 95% CI: 1.28–4.49); greater sagittal range of motion (SROM) (OR = 2.00, 95% CI: 1.58–2.53); higher body mass index (BMI) (OR = 1.30, 95% CI: 1.18–1.42); advanced age (OR = 1.21, 95% CI: 1.12–1.30); and high-intensity postoperative activity (OR = 1.83, 95% CI: 1.23–2.44). Among the herniation types, sequestrated disc herniation was associated with the highest recurrence risk. No significant correlation was found between the Pfirrmann grading system and recurrence risk (OR = 1.28, 95% CI: 0.95–1.60).

**Conclusion:**

The results of this meta-analysis indicate that recurrence after PELD for LDH is associated with a range of factors. Significant independent patient-related risk factors include advanced age, higher BMI, smoking, diabetes, and the presence of Modic changes (especially type II). Regarding surgical factors, intraoperative annulus fibrosus rupture significantly increases the risk of recurrence. Postoperatively, engaging in high-intensity activities too early or having a greater lumbar SROM also markedly elevates the probability of recurrence.

## 1 Introduction

Lumbar disc herniation (LDH) is a common pathological condition of the lumbar spine, typically caused by herniated disc material irritating and/or compressing nerve roots, leading to a series of symptoms such as low back pain, radiating leg pain, and numbness (1). Epidemiological studies indicate that the lifetime risk of developing LDH is approximately 30% (2). In recent years, percutaneous endoscopic lumbar discectomy (PELD) has emerged as a major minimally invasive technique for treating LDH due to its advantages of minimal trauma, reduced blood loss, and faster postoperative recovery (3). It is strongly recommended (Grade 1 recommendation) in the Clinical Guidelines for the Diagnosis and Treatment of Lumbar Disc Herniation (1). PELD effectively relieves nerve root compression and alleviates radicular symptoms by removing the herniated nucleus pulposus (3). However, due to factors such as its steep learning curve, patient selection criteria, and technical aspects of the puncture procedure, PELD carries a certain risk of postoperative recurrence (4). Existing research suggests that the recurrence rate of LDH at the L4/L5 level after PELD can reach 7%, associated with factors like intervertebral disc degeneration, fat infiltration of paraspinal muscles, and lumbar sagittal range of motion (5). Some domestic studies have identified age, history of diabetes, duration of symptoms, work-related physical strain, and degree of disc degeneration as independent risk factors for recurrence (6). Other studies suggest that Pfirrmann grade, body mass index (BMI), age, and postoperative intervertebral range of motion are also significantly correlated with recurrence (7). Currently, findings regarding the risk factors for recurrence after PELD exhibit some heterogeneity, and there is a lack of comprehensive systematic evaluation. Therefore, this study employs a meta-analysis approach to systematically evaluate the risk factors associated with recurrence following PELD for LDH, aiming to provide an evidence-based foundation for identifying high-risk populations and guiding clinical interventions.

## 2 Data and methods

### 2.1 Literature search strategy

A systematic computerized search was conducted in the following databases: PubMed, Cochrane Library, EMbase, China National Knowledge Infrastructure (CNKI), WanFang Database, and VIP Database. The search timeframe spanned from the inception of each database to August 30, 2025. The search strategy was developed and refined based on the filtering strategy formulated by Geersing et al. for systematic reviews of prediction models. The English search terms included: “*lumbar disc herniation*”, “*percutaneous endoscopic lumbar discectomy*”, “*percutaneous endoscopic transforaminal discectomy*”, “*percutaneous endoscopic interlaminar discectomy*”, “*endoscopy*”, “*foraminoscopy*”, “*risk prediction*”, “*risk assessment*”, “*prediction model*”, “*prognostic model*”, “*decision support*”, among others. There were no restrictions on the country or region of publication. The language was restricted to Chinese and English.

### 2.2 Search strategy example (PubMed)

Using PubMed as an example, the detailed search strategy is presented in Table 1.

**Table 1:** Search strategy for the PubMed database.

No.	Search strategy
#1	lumbar disc herniation[Title/Abstract] OR lumbar disk herniation[Title/Abstract] OR lumbar disc protrusion[Title/Abstract] OR lumbar herniated disc[Title/Abstract] OR lumbar herniated disk[Title/Abstract] OR lumbar intervertebral disc displacement[MeSH Terms] OR lumbar intervertebral disc herniation[Title/Abstract] OR lumbar intervertebral disc prolapse[Title/Abstract] OR lumbar intervertebral disk herniation[Title/Abstract] OR LDH[Title/Abstract]
#2	percutaneous endoscopic lumbar discectomy[Title/Abstract] OR percutaneous endoscopic lumbar diskectomy[Title/Abstract] OR percutaneous transformational endoscopic discectomy[Title/Abstract] OR percutaneous endoscopic transformational discectomy [Title/Abstract] OR percutaneous interlaminar endoscopic discectomy[Title/Abstract] OR percutaneous endoscopic interlaminar discectomy[Title/Abstract] OR endoscopic transformational discectomy [Title/Abstract] OR endoscopic interlaminar discectomy [Title/Abstract] OR PELD[Title/Abstract] OR PTED[Title/Abstract] OR PIED [Title/Abstract] OR TESSYS[Title/Abstract] OR Yeung endoscopic spine system [Title/Abstract]
#3	recurrence[MeSH Terms] OR recurrent[Title/Abstract] OR recurrence[Title/Abstract] OR relapse[Title/Abstract] OR failed back surgery syndrome[Title/Abstract] OR FBSS[Title/Abstract] OR reoperation[MeSH Terms] OR reoperation[Title/Abstract] OR residual[Title/Abstract]
#4	risk factors[MeSH Terms] OR predictive value of tests[MeSH Terms] OR prognosis[MeSH Terms] OR odds ratio[MeSH Terms] OR multivariate analysis[MeSH Terms] OR logistic models[MeSH Terms] OR retrospective studies[MeSH Terms] OR prospective studies[MeSH Terms] OR cohort studies[MeSH Terms] OR case-control studies[MeSH Terms] OR risk factor[Title/Abstract] OR predictive factor[Title/Abstract] OR prognostic factor[Title/Abstract] OR predictor [Title/Abstract] OR associated factor[Title/Abstract] OR correlation[Title/Abstract] OR regression analysis[Title/Abstract] OR univariate[Title/Abstract] OR multivariate [Title/Abstract] OR nomogram[Title/Abstract] OR scoring system[Title/Abstract]
#5	#1 AND #2 AND #3 AND #4

Additionally, the reference lists of the included studies were manually screened to identify further potentially relevant publications.

### 2.3 Inclusion and exclusion criteria

#### 2.3.1 Inclusion criteria

(i) Study participants were patients with LDH who underwent PELD, including both the PTED and PIED approaches. (ii) The study focused on the development or validation of a risk prediction model for recurrence in LDH patients following PELD. (iii) The study design was a cohort study or a case-control study. (iv) The primary outcome was recurrent lumbar disc herniation. (v) The patient follow-up duration was at least 6 months.

#### 2.3.2 Exclusion criteria

(i) Studies that only investigated independent risk factors without developing a complete prediction model. (ii) Reviews, commentaries, conference abstracts, dissertations, or duplicate publications. (iii) Models developed solely based on the results of systematic reviews or meta-analyses. (iv) Prediction models containing fewer than 2 predictors. (v) Studies for which the full text was unavailable or contained insufficient information.

### 2.4 Data extraction

The literature screening and data extraction were conducted independently by two investigators. The collected data were cross-checked between them. In case of any disagreement, a third investigator was consulted to reach a consensus. The screening process was performed in two stages: first, an initial screening based on the titles and abstracts to exclude irrelevant records; second, a full-text review of the remaining articles to determine final eligibility. Data were extracted using a checklist based on the critical appraisal and data extraction requirements for systematic reviews of prediction model studies. The extracted information included: first author, year of publication, country, study design, data source, baseline characteristics of the study population, sample size, time of measurement, candidate variables, predicted outcome, model development method, internal validation method, approach for handling missing data, model presentation format, model predictive performance, and the final set of predictors included in the model. If multiple models were presented within a single included study, data were extracted separately for each model.

### 2.5 Quality assessment (risk of bias)

The methodological quality and risk of bias of the included studies were assessed using the Newcastle-Ottawa Scale (NOS), as recommended by the Cochrane Collaboration. The NOS evaluates studies across eight items within three domains: selection of study groups, comparability of groups, and ascertainment of the outcome of interest. The total possible score is 9 points. Studies scoring ≥7 points, 6 points, and ≤5 points were classified as high, moderate, and low quality, respectively.

### 2.6 Outcome measures

The primary outcome measures extracted from the included studies encompassed the following variables:

- Patient Characteristics: gender, age, BMI.
- Clinical and Comorbidity Factors: diabetes mellitus, smoking status, postoperative activity intensity.
- Imaging Characteristics: type of lumbar disc herniation, Modic changes, Pfirrmann grade, nucleus pulposus degeneration grade, sagittal range of motion.
- Surgical Factor: intraoperative annulus fibrosus rupture.

### 2.7 Statistical analysis

Meta-analysis was performed using Stata 12.0 software. The pooled effect estimates were calculated with odds ratios (OR) serving as the pooled statistic, and each effect size was expressed with its 95% confidence interval (CI). Heterogeneity among the included studies was assessed using the Chi-squared test (with a significance level of *α*=0.1) and the *I^2^* statistic. If the Chi-squared test resulted in *P* > 0.10 and *I^2^* ≤ 50%, indicating acceptable heterogeneity, a fixed-effects model was applied for the pooled analysis. Conversely, if *P* ≤ 0.10 or *I^2^* > 50%, suggesting significant heterogeneity, the sources of heterogeneity were explored. Following this exploration, a decision was made on whether to utilize a random-effects model for pooled analysis and to conduct subgroup analyses. Within the meta-analysis, a *P*-value < 0.05 was considered statistically significant for comparisons between groups. The stability of the pooled results was evaluated by altering the statistical model. If the results were deemed unstable, a sensitivity analysis was further performed using the leave-one-out method. Finally, publication bias among the included studies was assessed using Egger's test and the trim-and-fill method.

## 3 Results

### 3.1 Literature search results and screening flow

**Figure 1:**
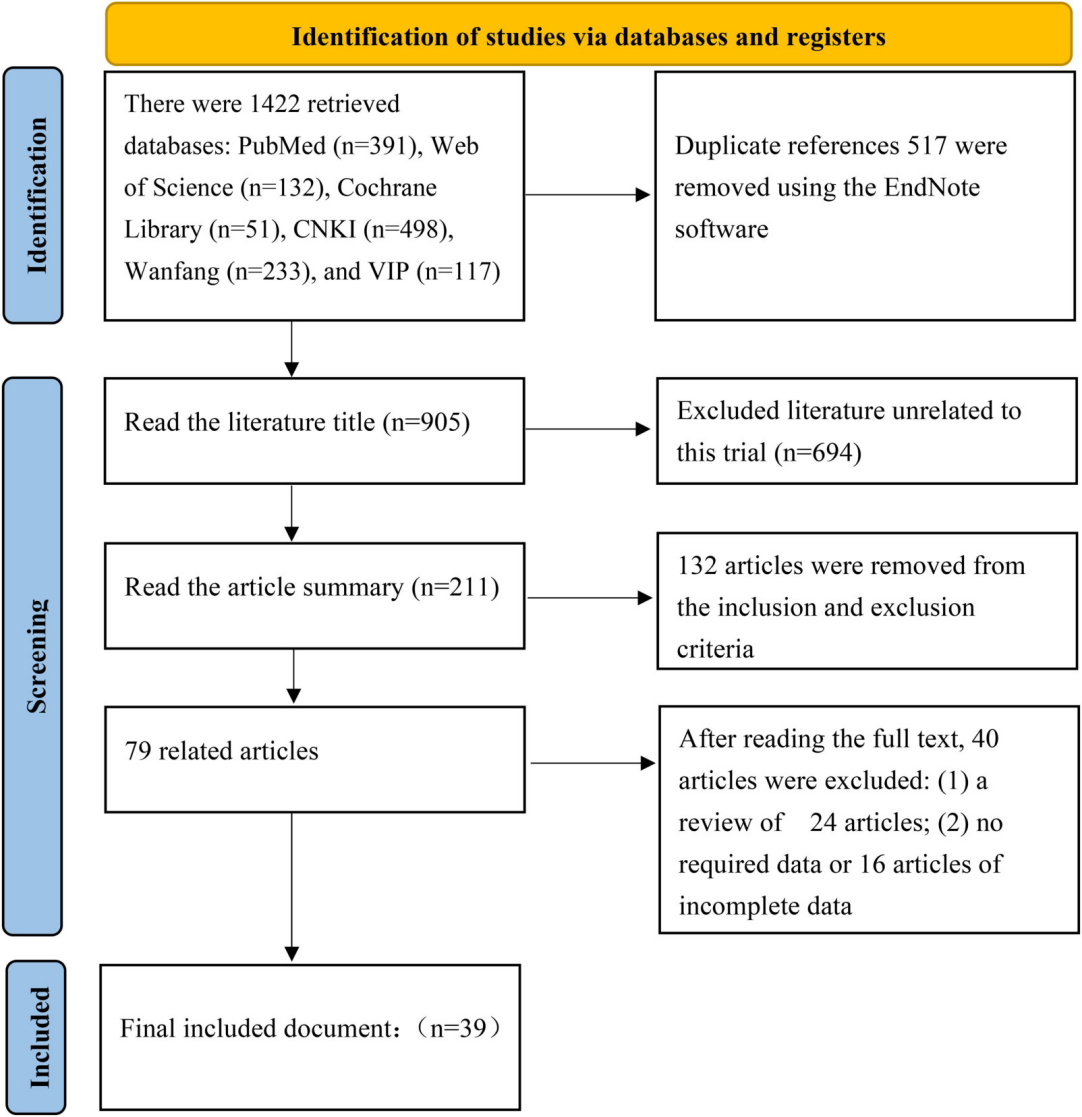
Literature screening process and results.

A total of 1,422 records were initially identified through the database searches: 391 from PubMed, 132 from Web of Science, 51 from the Cochrane Library, 498 from the CNKI, 233 from the WanFang Database, and 117 from the VIP Database. After the final screening stage, 39 studies meeting the eligibility criteria were included. All 39 studies were case-control studies. A total of 14,454 patients with lumbar disc herniation were included in this meta-analysis. The flow diagram detailing the number of records at each stage of the screening process is presented in Figure 1. The baseline characteristics of the 39 included studies are summarized in Table 2.

**Table 2:** Basic characteristics of the 39 studies included.

Author (year)	Country	Study type	Total patients	Recurrence cases	Recurrence rate(%)	Age (years)	Follow-up/month	Surgical approach	Risk factors assessed
Liang ZL 2020 (8)	China	CC	168	17	10.12	42.51 ± 14.83	6	PTED	②④⑧⑨
Qi WB 2019 (9)	China	CC	136	19	13.97	39–75 (63.7 ± 5.8)	12	PTED	⑥⑦⑪
Liu X 2019 (10)	China	CC	128	26	20.31	45.46	>3	PTED	⑦
Zhang YS 2019 (11)	China	CC	285	11	3.86	25–62 (43.07 ± 5.44)	>6	PTED	②④⑤⑦⑧⑩⑫
Wei B 2019 (12)	China	CC	130	13	10.00	38–74 (51.37 ± 6.10)	12	PTED	⑤⑦⑩⑪
Chen HL 2019 (13)	China	CC	212	12	5.66	18–86	≥24	PTED	③
Xu JT 2019 (14)	China	CC	292	16	5.48	39.98	24	PTED	③
Li Y 2018 (15)	China	CC	378	12	3.17	24–84 (52.3 ± 3.6)	30	PTED	②⑤
Kim JM 2007 (16)	Korea	CC	84	42	50.00	18–76 (47.4)	/	PTED	②③⑦
He H 2023(a) (17)	China	CC	690	63	9.13	43.66 ± 14.60	24	PELD	③⑫
Wang F 2022 (18)	China	CC	942	57	6.05	42.1	≥12	PELD	①⑦⑧⑨
Sic A 2022 (19)	China	CC	396	46	11.62	45 ± 13	≥12	PELD	⑧⑨
Shi H 2021 (20)	China	CC	204	68	33.33	46.21 ± 9.63	13–28	PELD	⑥⑩
Zhao C 2021 (21)	China	CC	181	23	12.71	23–79 (52.1 ± 13.7)	≥12	PELD	③⑫
Ding Y 2020 (22)	China	CC	599	33	5.51	55.58 ± 11.98	≥12	PELD	④⑥⑦⑧⑨
Kong M 2020 (23)	China	CC	654	46	7.03	55.4 ± 12.2	27.6 ± 8.2	PELD	④⑦⑧⑨⑩
Yu C 2020 (24)	China	CC	484	46	9.50	52.978 ± 6.962	12–48	PELD	⑦⑨⑩⑫
Belykh E 2017 (25)	Russia	CC	350	50	14.29	43.6 ± 7.9 (30–61)	≥36	PELD	⑧
Yao Y 2017 (26)	China	CC	3,378	116	3.43	16–89 (47.5)	≥24	PELD	②⑦⑨
Yao L 2016 (27)	China	CC	708	111	15.68	46(13–79 )	12	PELD	③⑦⑨
Shi M 2013 (28)	Iran	CC	160	40	25.00	45.82 ± 11.23	18	PELD	①⑧
Kim K 2009 (29)	Korea	CC	157	14	8.92	15–78 (45.2 ± 14.2)	24–108	PELD	⑧⑩⑫
He H 2023 (30)	China	CC	690	46	6.67	43.44 ± 14.65	>6	PELD	⑦⑨
Zhu L 2025 (31)	China	CC	145	26	17.93	43–70.5	6	PELD	⑦⑫
Zhao ZJ 2023 (32)	China	CC	110	11	10.00	/	12	PELD	⑤⑥⑪ ⑫
Zou YF 2020 (33)	China	CC	908	52	5.73	46.32 ± 11.63	12	PELD	②⑧⑨
Zhang GQ 2021 (34)	China	CC	230	29	12.61	/	6	PTED	②④⑦⑧⑨
Wang SY 2024 (35)	China	CC	300	38	12.67	51.84 ± 12.93	>6	PTED	④⑨
Fang FZ 2021 (36)	China	CC	324	29	8.95	/	6	PTED	④⑦⑧
Dan ZM 2023 (37)	China	CC	171	57	33.33	45.63 ± 14.70	>6	PELD	②③⑫
Dong JG 2021 (38)	China	CC	200	25	12.50	19–76 (45.06 ± 13.56)	12	PEID	⑥⑦
Ju SL 2023 (39)	China	CC	189	19	10.05	50.39 ± 13.78	19.74 ± 9.31	PELD	③⑧⑨
Li YL 2022 (40)	China	CC	186	20	10.75	34∼78	12∼24	PTED	②⑤⑦⑨⑪
Li ZP 2022 (41)	China	CC	285	19	6.67	13–90(54.9 ± 12.8)	30.7	PTED	③⑪
Liang X 2024 (42)	China	CC	286	44	15.38	14∼85	36	PELD	③⑨⑫
Ma QL 2022 (43)	China	CC	942	57	6.05	41.2 ± 11.4	12	PELD	①⑦⑧⑨
Zhang P 2019(a) (44)	China	CC	512	41	8.01	16–82(47.10 ± 16.72)	1∼44	PELD	③⑦⑨
Zhang P 2019(b) (45)	China	CC	476	36	7.56	14–87(47.4 ± 17.4)	1∼48	PELD	③⑦⑨
Zhao C 2022 (46)	China	CC	1,210	62	5.12	52.1 ± 13.7	12	PTED	③⑫

① Gender; ② Type of lumbar disc herniation; ③ Modic changes; ④ Diabetes mellitus; ⑤ Postoperative activity intensity; ⑥ Pfirrmann grade; ⑦ Age; ⑧ Smoking; ⑨ Body mass index (BMI); ⑩ Nucleus pulposus degeneration grade; ⑪ Intraoperative annulus fibrosus rupture; ⑫ Sagittal range of motion; PELD, percutaneous endoscopic lumbar discectomy; PTED, percutaneous transforaminaendoscopic discectomy; PIED, percutaneous interlaminar endoscopic discectomy; CC, case-control study.

### 3.2 Data extraction and quality assessment of included studies

**Figure 2:**
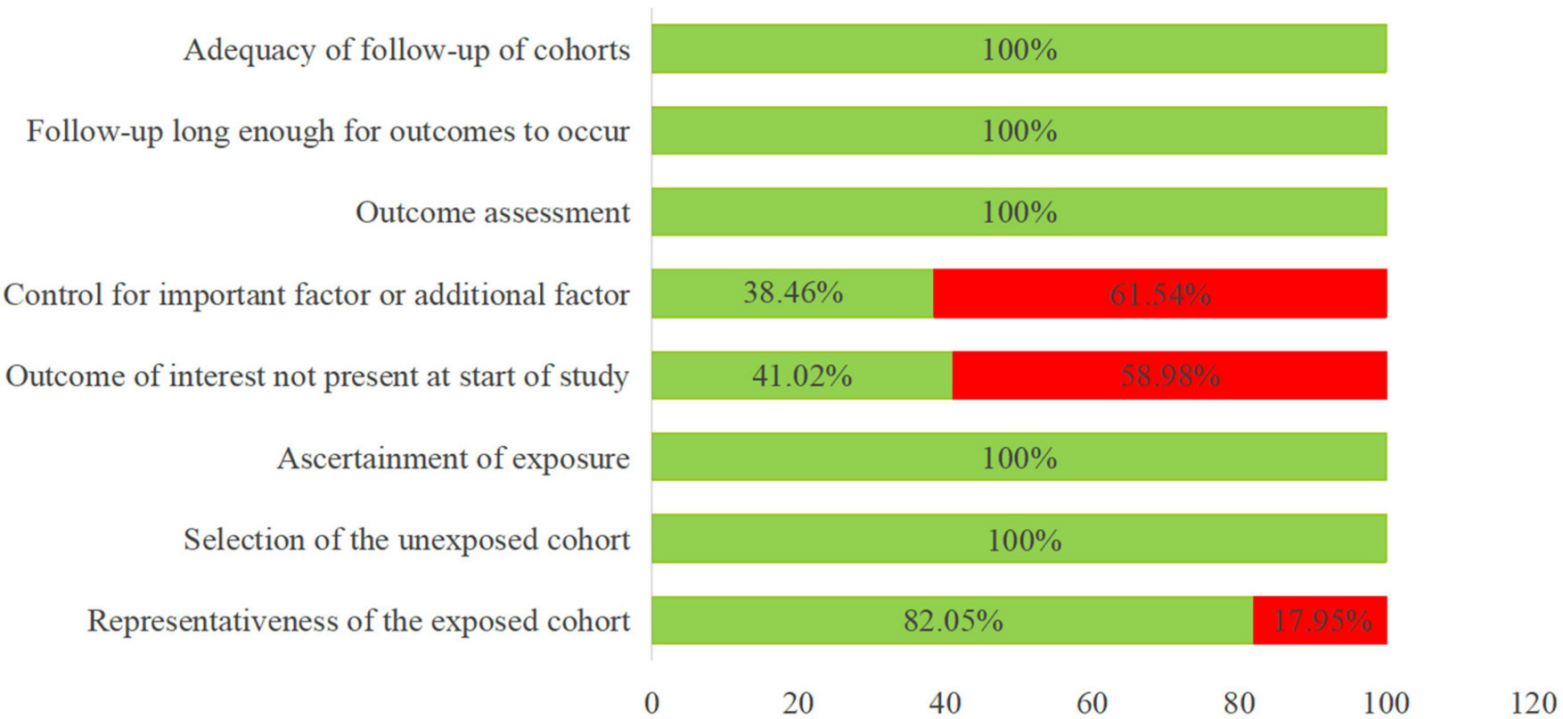
Include in the study quality assessment diagram. Compliance rates for each methodological item of the NOS.

**Figure 3:**
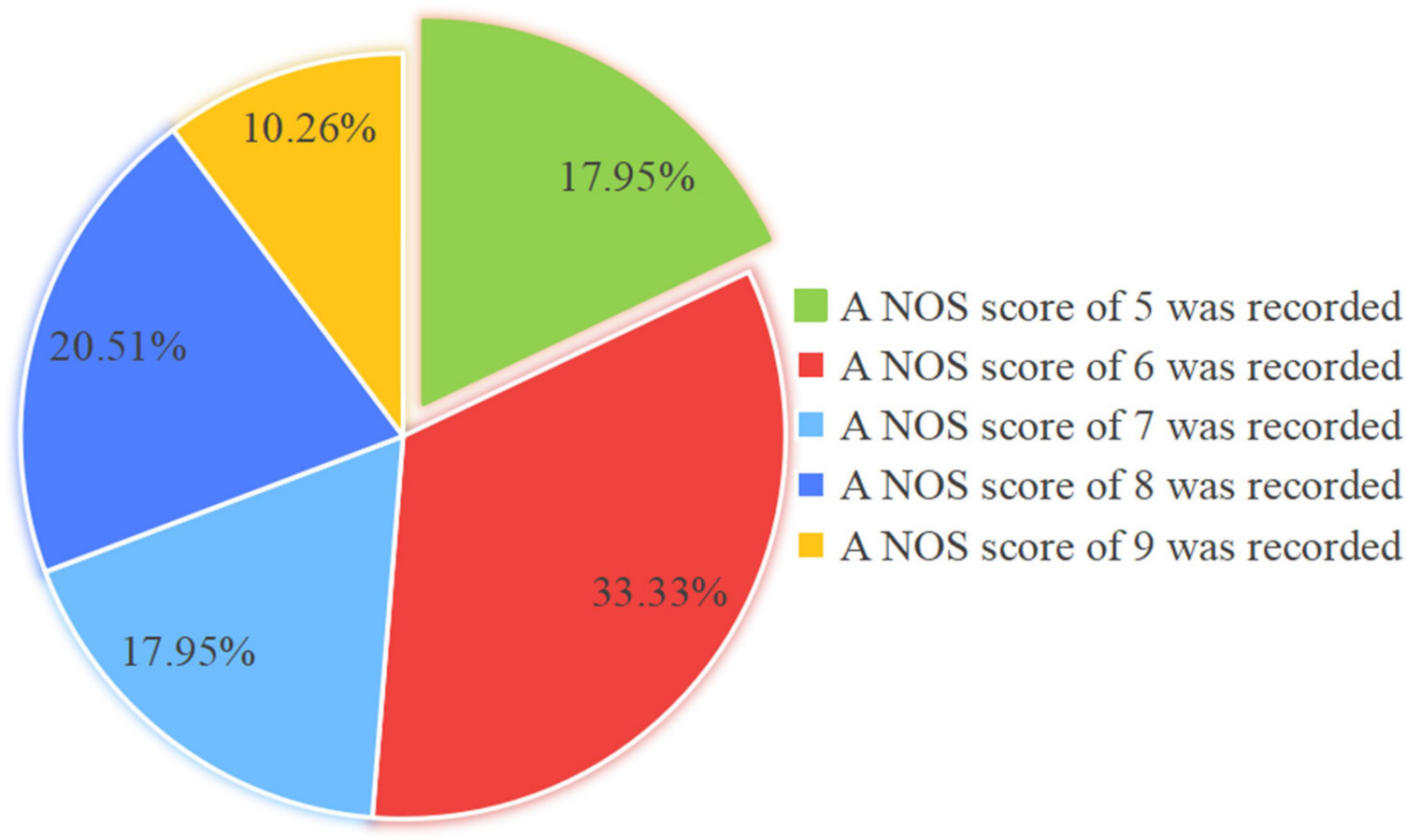
Percentage distribution of NOS scores. Overall distribution of NOS scores.

Two independent reviewers evaluated the methodological quality of the included studies using the NOS. Among the included studies, seven were rated as high quality and four as moderate quality. The quality assessments are detailed in Table 3 and Figure 2, while the basic characteristics of the studies are presented in Table 2. Figure 2 presents the compliance rates of the 39 included studies across the specific methodological items of the NOS. All studies (100%) adequately fulfilled the criteria for “*representativeness of the exposed cohort*,” “*adequacy of follow-up duration*,” and “assessment of outcome.” However, compliance was notably low (38.46%) for the critical item “control for important confounding factors,” indicating that most studies failed to adequately identify or adjust for potential confounding variables, which represents a major methodological limitation. Figure 3 displays the overall distribution of NOS scores. Studies with a score of 6 points constituted the largest proportion (33.33%). Nearly half of the studies (48.72%) were rated as high quality (score ≥ 7). This distribution suggests that the body of evidence integrated in this meta-analysis is of moderate overall methodological quality; however, the potential bias introduced by studies with moderate and low-quality ratings must be considered when interpreting the results.

**Table 3:** General diagram of quality assessment of the included literature.

First author	Total	Representativeness of the exposed cohort	Selection of the unexposed cohort	Ascertainment of exposure	Outcome of interest not present at start of study	Control for important factor or additional factor	Outcome assessment	Follow-up long enough for outcomes to occur	Adequacy of follow-up of cohorts
Liang ZL 2020 (8)	6	★	★	★			★	★	★
Qi WB 2019 (9)	7	★	★	★	★		★	★	★
Liu X 2019 (10)	6	★	★	★			★	★	★
Zhang YS 2019 (11)	7	★	★	★	★		★	★	★
Wei B 2019 (12)	7	★	★	★		★	★	★	★
Chen HL 2019 (13)	6	★	★	★			★	★	★
Xu JT 2019 (14)	8	★	★	★	★	★	★	★	★
Li Y 2018 (15)	6	★	★	★			★	★	★
Kim JM 2007 (16)	8	★	★	★	★	★	★	★	★
He H 2023(a) (17)	5		★	★			★	★	★
Wang F 2022 (18)	6	★	★	★			★	★	★
Sic A 2022 (19)	8	★	★	★	★	★	★	★	★
Shi H 2021 (20)	9	★	★	★	★	★★	★	★	★
Zhao C 2021 (21)	8	★	★	★	★	★	★	★	★
Ding Y 2020 (22)	7	★	★	★	★		★	★	★
Kong M 2020 (23)	8	★	★	★	★	★	★	★	★
Yu C 2020 (24)	9	★	★	★	★	★★	★	★	★
Belykh E 2017 (25)	8	★	★	★	★	★	★	★	★
Yao Y 2017 (26)	9	★	★	★	★	★★	★	★	★
Yao L 2016 (27)	9	★	★	★	★	★★	★	★	★
Shi M 2013 (28)	8	★	★	★	★	★	★	★	★
Kim K 2009 (29)	7	★	★	★	★		★	★	★
He H 2023(b) (30)	6	★	★	★			★	★	★
Zhu L 2025 (31)	5		★	★			★	★	★
Zhao ZJ 2023 (32)	5		★	★			★	★	★
Zou YF 2020 (33)	6	★	★	★			★	★	★
Zhang GQ 2021 (34)	5		★	★			★	★	★
Wang SY 2024 (35)	5		★	★			★	★	★
Fang FZ 2021 (36)	6	★	★	★			★	★	★
Dan ZM 2023 (37)	6	★	★	★			★	★	★
Dong JG 2021 (38)	6	★	★	★			★	★	★
Ju SL 2023 (39)	6	★	★	★			★	★	★
Li YL 2022 (40)	6	★	★	★			★	★	★
Li ZP 2022 (41)	5		★	★			★	★	★
Liang X 2024 (42)	7	★	★	★		★	★	★	★
Ma QL 2022 (43)	7	★	★	★		★	★	★	★
Zhang P 2019(a) (44)	5		★	★			★	★	★
Zhang P 2019(b) (45)	8	★	★	★	★	★	★	★	★
Zhao C 2022 (46)	6	★	★	★			★	★	★

One star (★) represents one point, with a maximum score of 9 points. Studies scoring ≥7 points, 6 points, and ≤5 points were classified as high-quality, moderate-quality, and low-quality studies, respectively.

### 3.3 Results of the meta-analysis

#### 3.3.1 Recurrence rate of lumbar disc herniation

**Figure 4:**
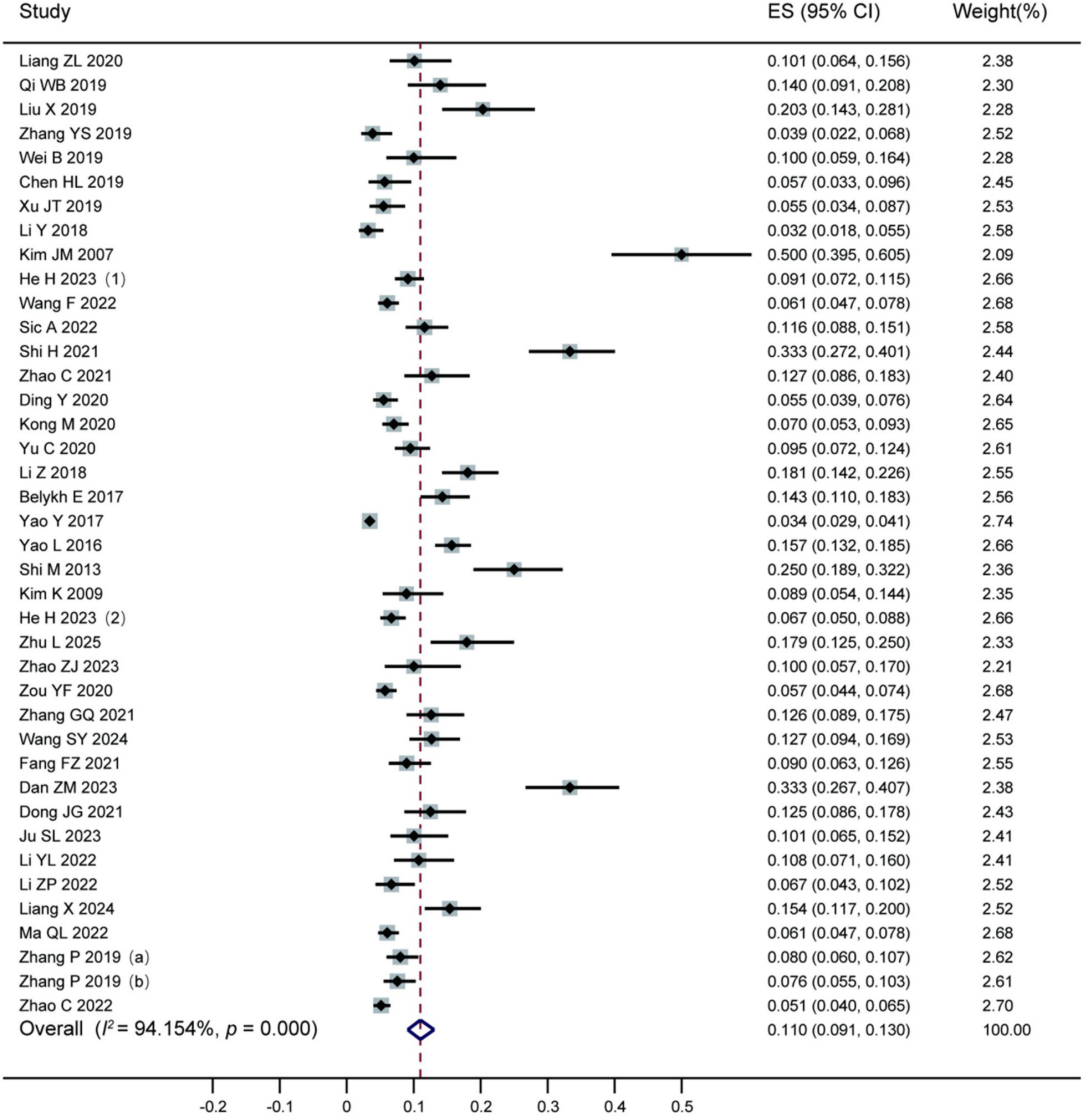
Forest plot of the recurrence rate after percutaneous endoscopic lumbar discectomy. The pooled recurrence rate following percutaneous endoscopic lumbar discectomy for lumbar disc herniation was 11%, based on data from 39 included studies.

A total of 39 studies included in this meta-analysis reported the effect size (ES) and its 95% CI for the postoperative recurrence rate of lumbar disc herniation following percutaneous endoscopic lumbar discectomy. The individual effect sizes and their corresponding weights from each study are detailed in the accompanying table. The pooled analysis showed an overall effect size of 0.110 (95% CI: 0.091–0.130), indicating a pooled recurrence rate of 11.0%. Significant heterogeneity was observed among the studies (*I^2^* = 94.154%, *P* = 0.000), as illustrated in Figure 4.

#### 3.3.2 Influence of gender on recurrence

**Figure 5:**
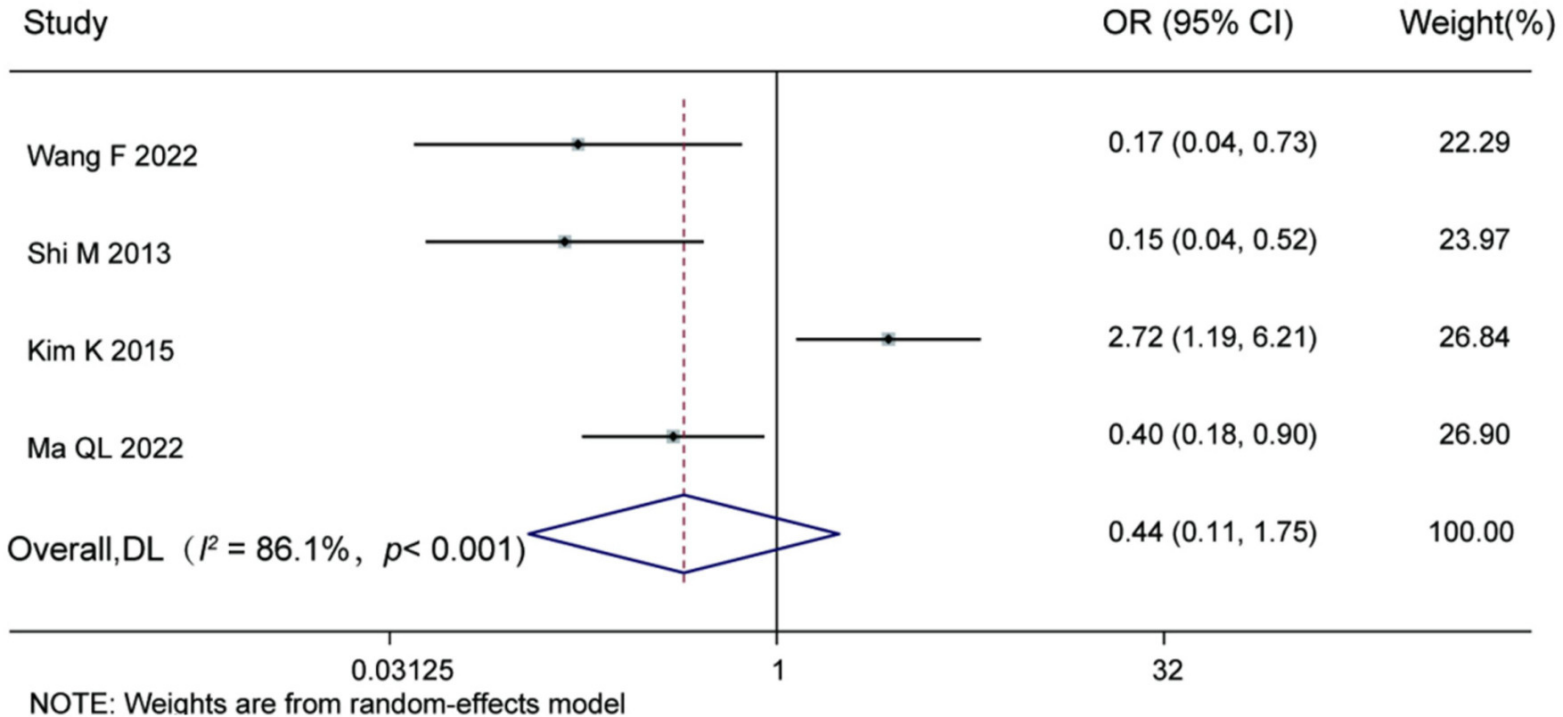
Forest plot of the association between gender and recurrence.

Four studies reported data on patient gender. Substantial heterogeneity was observed among these studies (*I^2^* = 86.1%, Cochran's *Q* test *P* < 0.001), prompting the use of a random-effects model for the meta-analysis. The results indicated no statistically significant association between gender and the risk of postoperative recurrence (OR = 0.44, 95% CI: 0.11–1.75, *P* = 0.24). For details, see Figure 5.

#### 3.3.3 Influence of age on recurrence

**Figure 6:**
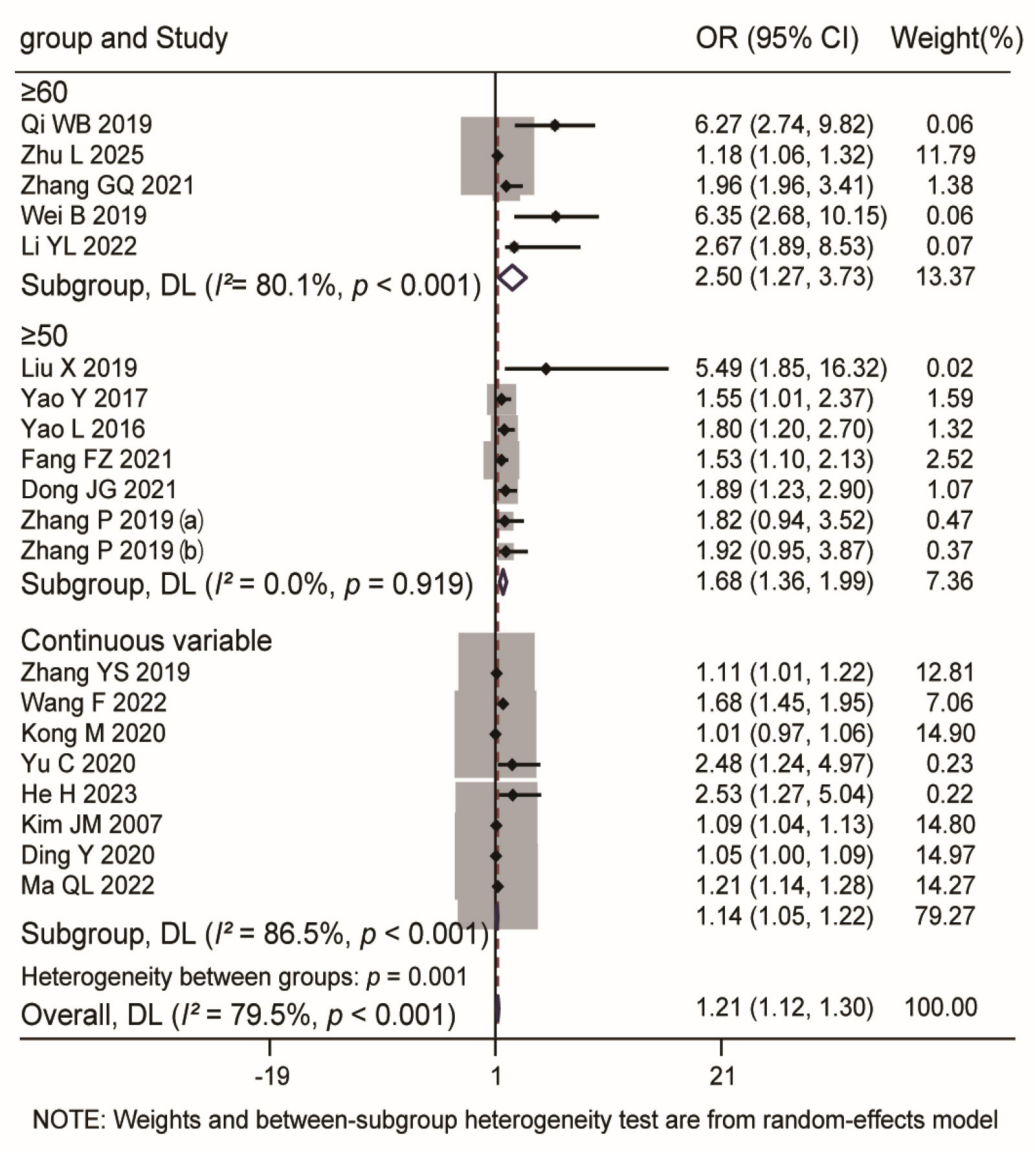
Forest plot of the association between age and recurrence.

A total of 20 studies investigating the association between age and postoperative recurrence were included in the analysis using a random-effects model. The overall pooled analysis demonstrated a statistically significant association, with a pooled OR of 1.21 (95% CI: 1.12–1.30, *P* < 0.001), indicating that older age is a significant risk factor for recurrence. Subgroup analyses were performed based on the method of age measurement: In the ≥60 years subgroup (5 studies), significant heterogeneity was observed (*I^2^* = 80.1%, *P* < 0.001), and the pooled OR was 2.50 (95% CI: 1.27–3.73). In the ≥50 years subgroup (7 studies), no heterogeneity was detected (*I^2^* = 0.0%, *P* = 0.919), and the pooled OR was 1.68 (95% CI: 1.36–1.99). In the continuous variable subgroup (8 studies), significant heterogeneity was present (*I^2^* = 86.5%, *P* < 0.001), and the pooled OR was 1.14 (95% CI: 1.05–1.22). The test for subgroup differences indicated that the method of age categorization significantly influenced the results (*P* = 0.001). For details, see Figure 6.

#### 3.3.4 Influence of body mass index on recurrence

**Figure 7:**
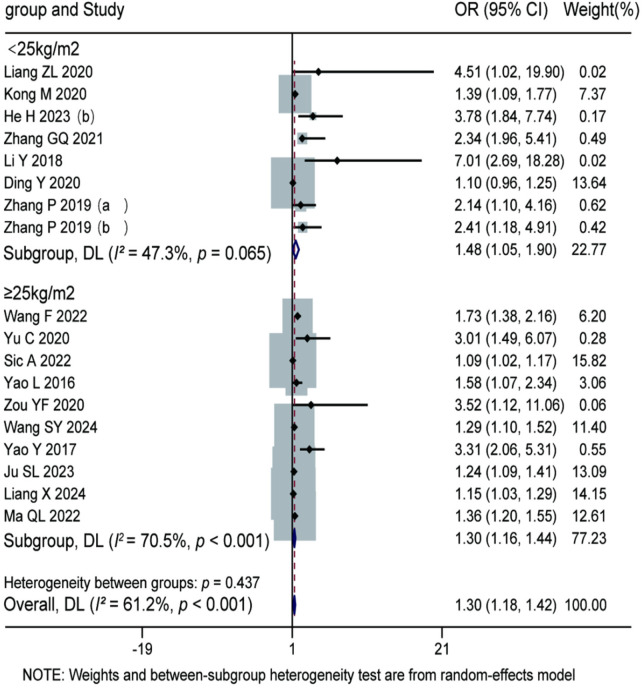
Forest plot of the association between body mass index and recurrence.

A total of 18 studies were included to investigate the influence of BMI on recurrence after percutaneous endoscopic lumbar discectomy using a random-effects model. The meta-analysis revealed a statistically significant overall pooled OR of 1.30 (95% CI: 1.18–1.42, *P* < 0.001), indicating that higher BMI is a significant risk factor for postoperative recurrence. Subgroup analysis based on BMI stratification showed: In the BMI < 25 kg/m^2^ subgroup (8 studies), the pooled OR was 1.48 (95% CI: 1.05–1.90), with low heterogeneity among studies (*I^2^* = 47.3%, *P* = 0.065). In the BMI ≥ 25 kg/m^2^ subgroup (10 studies), the pooled OR was 1.30 (95% CI: 1.16–1.44), with moderate heterogeneity observed (*I^2^* = 70.5%, *P* < 0.001). The test for subgroup differences showed no statistically significant effect of BMI categorization on the pooled effect size (*P* = 0.437). In conclusion, overweight and obesity (BMI ≥ 25 kg/m^2^) significantly increase the risk of recurrence after percutaneous endoscopic lumbar discectomy for lumbar disc herniation. For details, see Figure 7.

#### 3.3.5 Influence of lumbar disc herniation type on recurrence

**Figure 8:**
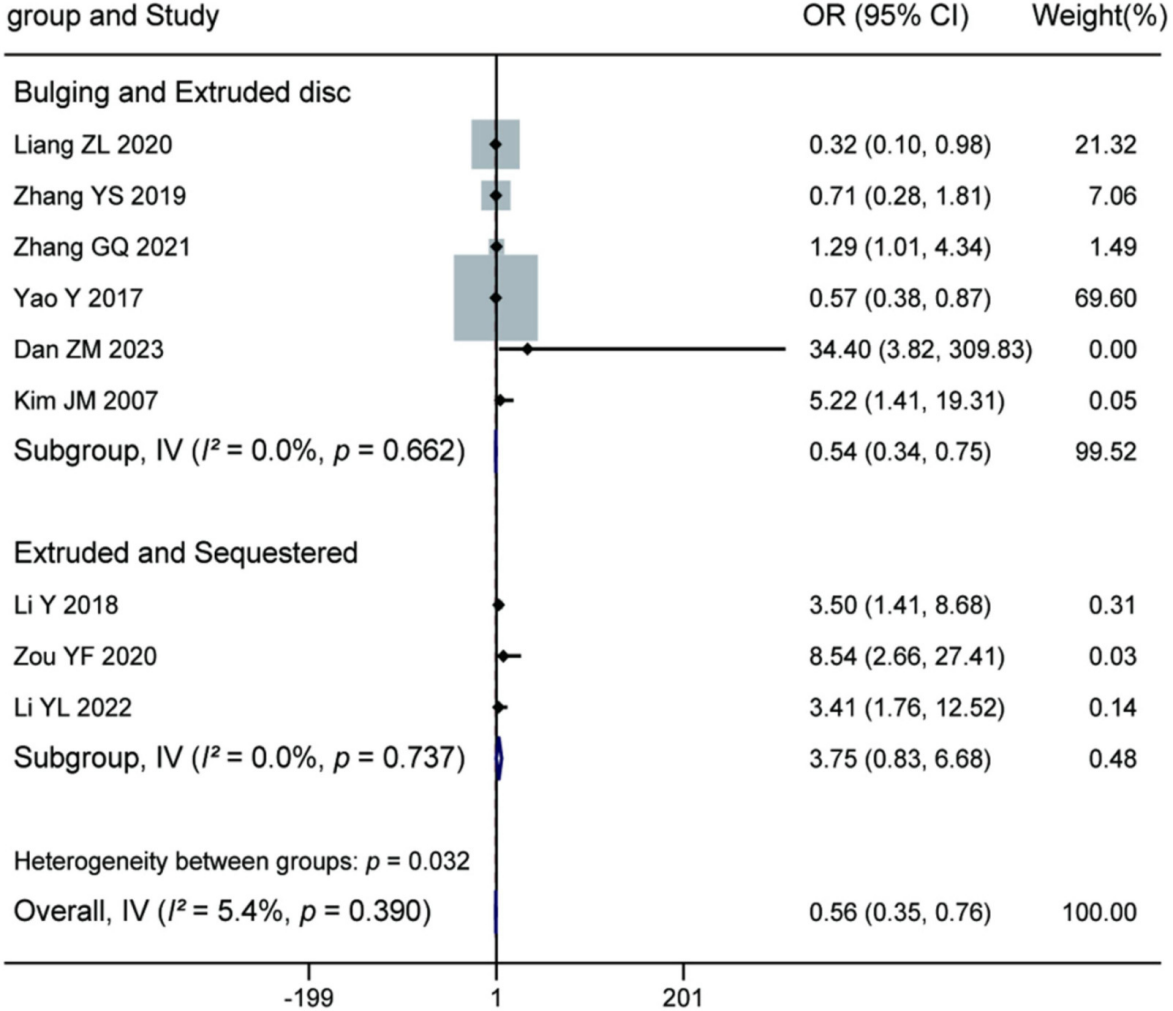
Forest plot of the association between lumbar disc herniation type and recurrence.

Meta-analysis of the 9 included studies demonstrated that the type of lumbar disc herniation was a significant factor influencing postoperative recurrence (pooled OR = 0.56, 95% CI: 0.35–0.76, *P* = 0.390). Subgroup analysis indicated that compared to protruded discs, bulging discs (6 studies) were associated with a significantly lower risk of recurrence (OR = 0.54, 95% CI: 0.34–0.75). Furthermore, in the comparison between protruded and sequestrated discs (3 studies), protruded discs showed a significantly lower recurrence risk than sequestrated discs (OR = 3.75, 95% CI: 0.83–6.68). The test for subgroup differences was statistically significant (*P* = 0.032), suggesting that more severe degrees of disc herniation are associated with higher postoperative recurrence risk, with sequestrated disc herniation carrying the highest risk. For details, see Figure 8.

#### 3.3.6 Influence of Modic changes on recurrence

**Figure 9:**
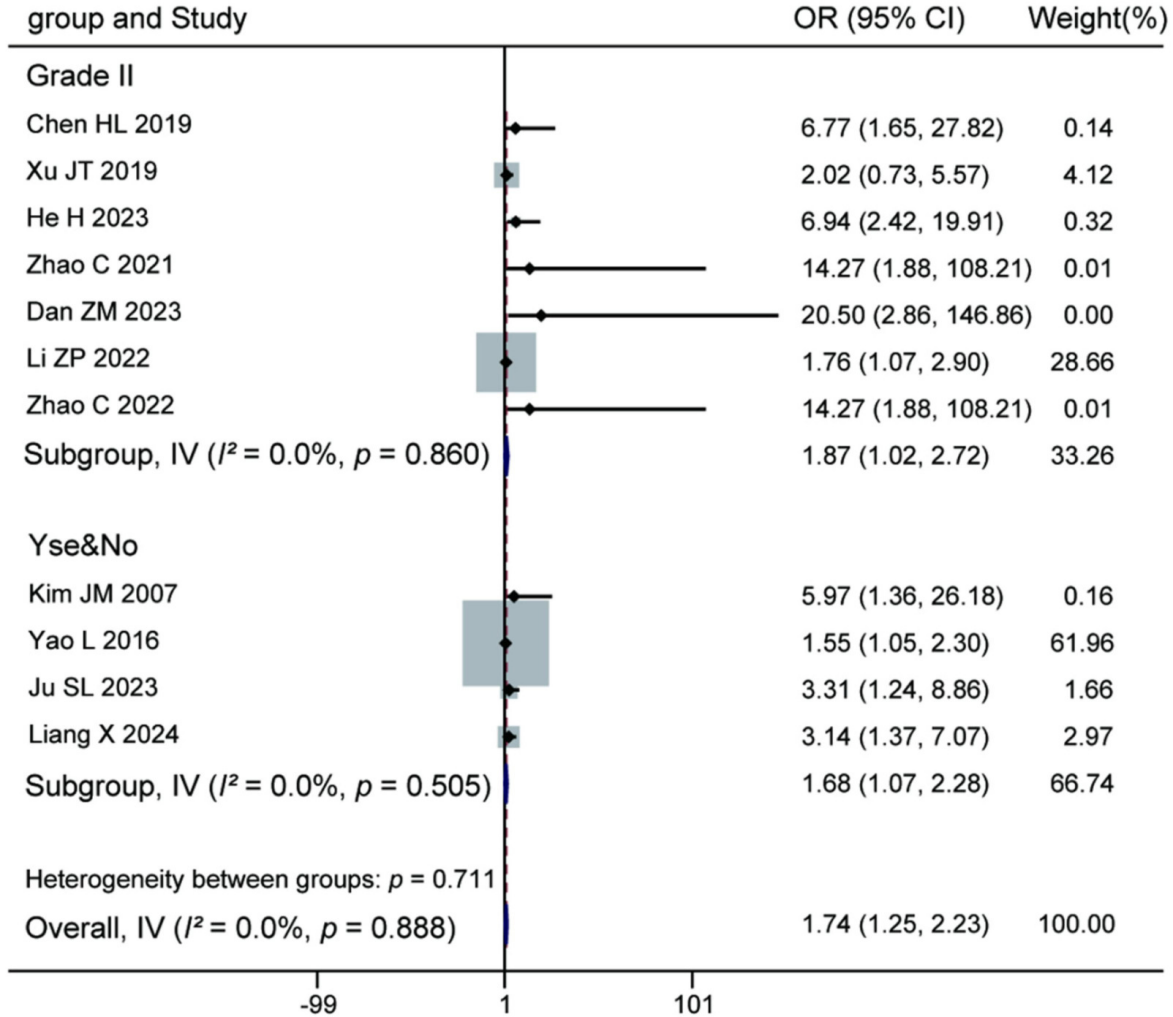
Forest plot of the association between Modic changes and recurrence.

Meta-analysis of the 11 included studies indicated that the presence of Modic changes was a risk factor for recurrence after percutaneous endoscopic lumbar discectomy (pooled OR = 1.74, 95% CI: 1.25–2.23, *P* = 0.888). Subgroup analysis showed that patients with Modic type II changes (7 studies) had a higher risk of recurrence (OR = 1.87, 95% CI: 1.02–2.72, *P* = 0.860). Similarly, the subgroup analysis based solely on the presence or absence of any Modic changes (4 studies) also suggested an increased recurrence risk (OR = 1.68, 95% CI: 1.07–2.28, *P* = 0.505). Heterogeneity was low in all subgroup and the overall analyses (*I^2^* = 0.0%). The test for subgroup differences was not statistically significant (*P* = 0.711). The stability of these results was satisfactory, suggesting that Modic changes, particularly type II, may increase the risk of postoperative recurrence. For details, see Figure 9.

#### 3.3.7 Influence of diabetes Mellitus on recurrence

**Figure 10:**
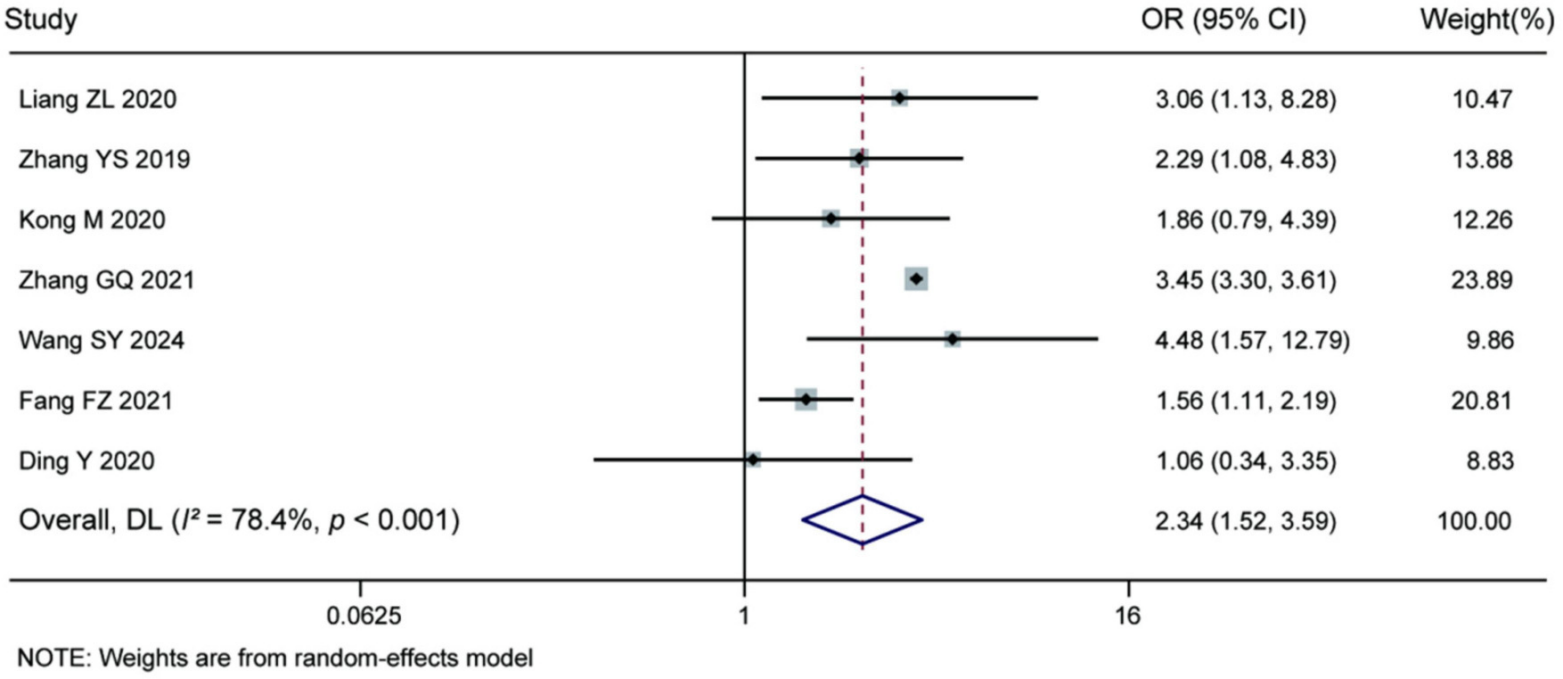
Forest plot of the association between diabetes mellitus and recurrence.

Meta-analysis of the 7 included studies demonstrated that diabetes mellitus was a significant risk factor for recurrence after percutaneous endoscopic lumbar discectomy (pooled OR = 2.34, 95% CI: 1.52–3.59, *P* < 0.001). Significant heterogeneity was observed among the studies (*I^2^* = 78.4%, *P* < 0.001), therefore a random-effects model was applied. The results indicate that patients with diabetes mellitus have a significantly higher risk of recurrence following percutaneous endoscopic lumbar discectomy compared to non-diabetic patients. For details, see Figure 10.

#### 3.3.8 Influence of postoperative activity intensity on recurrence

**Figure 11:**
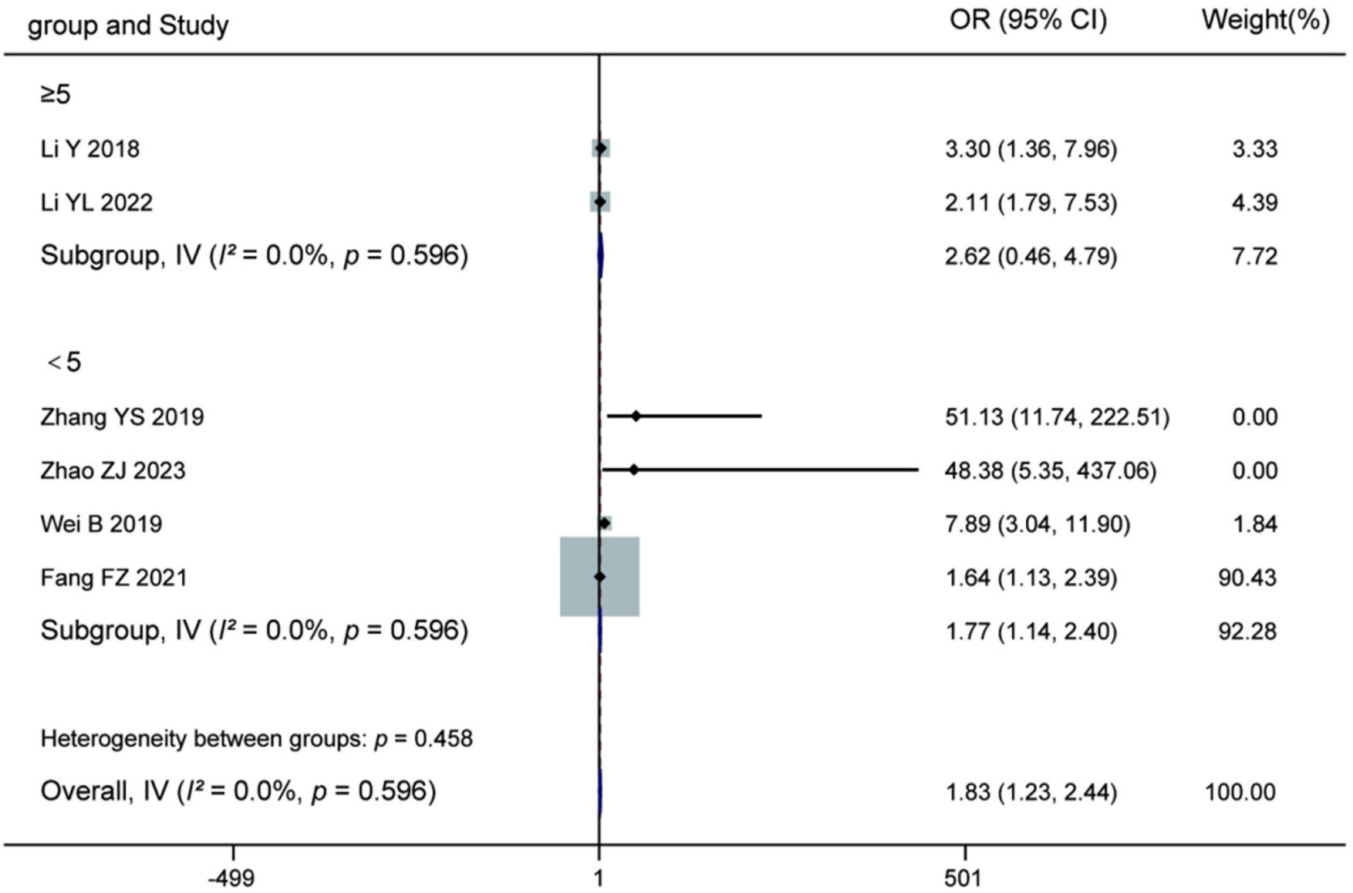
Forest plot of the association between postoperative activity intensity and recurrence.

Meta-analysis of the 6 included studies demonstrated that high-intensity postoperative activity was a significant risk factor for recurrence after percutaneous endoscopic lumbar discectomy (pooled OR = 1.83, 95% CI: 1.23–2.44, *P* = 0.596). Subgroup analysis indicated that regardless of the cut-off value used for defining activity intensity (≥5 level or <5 level), high-intensity activity was associated with increased recurrence risk. The risk was more pronounced in the subgroup using the <5 level cut-off (4 studies, OR = 1.77, 95% CI: 1.14–2.40). Heterogeneity among studies was acceptable (overall *I^2^* = 46.5%, *P* = 0.096), and a fixed-effects model was applied. The test for subgroup differences showed no statistical significance (*P* = 0.458). These results suggest that engaging in high-intensity activities too early during the postoperative period significantly increases recurrence risk, indicating that patients should be advised to appropriately control their activity intensity after surgery. For details, see Figure 11.

#### 3.3.9 Influence of Pfirrmann grading on recurrence

**Figure 12:**
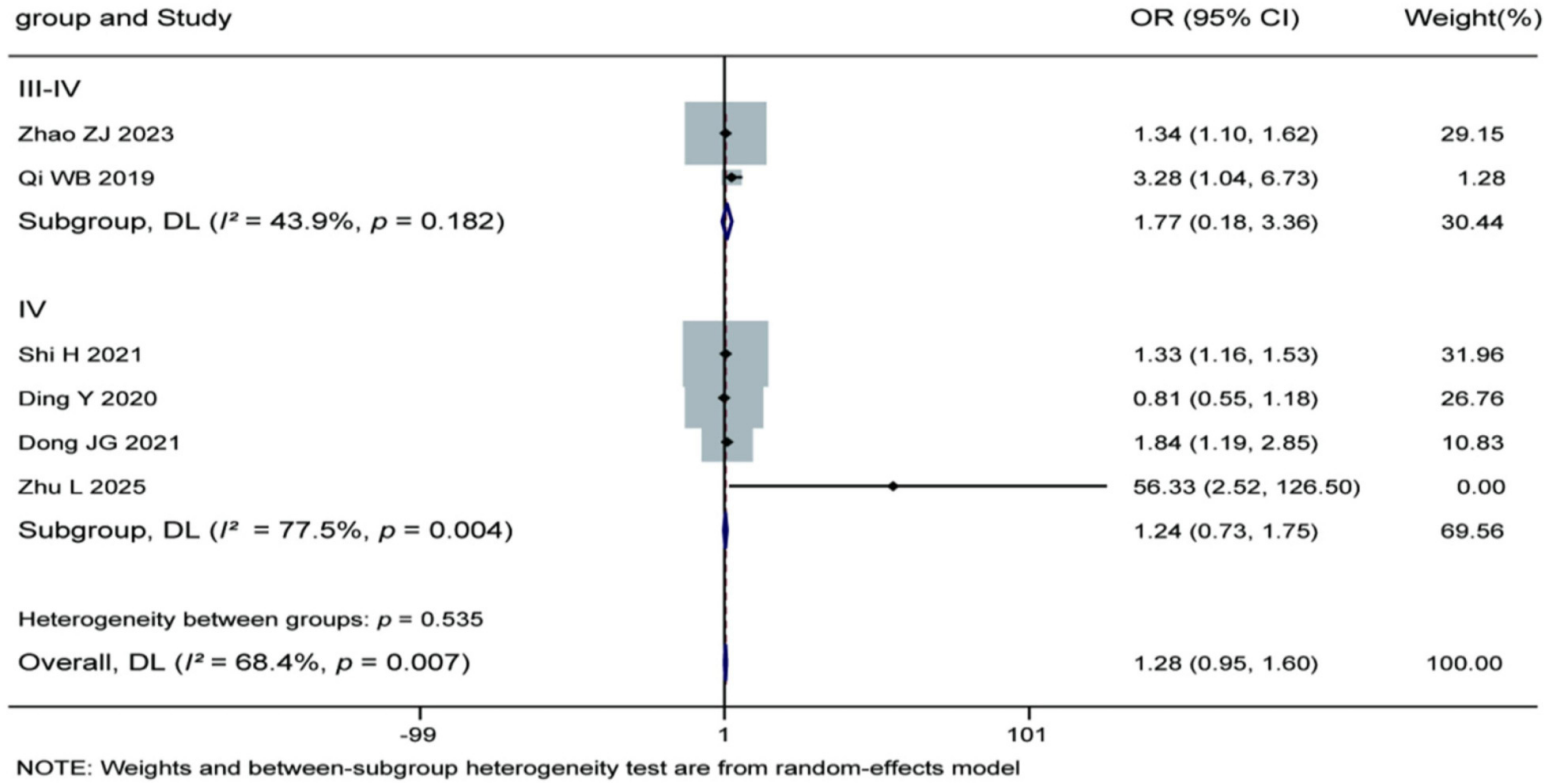
Forest plot of the association between Pfirrmann grading and recurrence.

Meta-analysis of the 6 included studies revealed no statistically significant association between intervertebral disc Pfirrmann grading and the risk of recurrence after percutaneous endoscopic lumbar discectomy (pooled OR = 1.28, 95% CI: 0.95–1.60). Subgroup analysis showed no significant differences in either the grades III-IV subgroup (2 studies; pooled OR = 1.77, 95% CI: 0.18–3.36) or the grade IV subgroup (4 studies; pooled OR = 1.24, 95% CI: 0.73–1.75). Moderate heterogeneity was observed among the studies (overall *I^2^* = 68.4%, *P* = 0.007), therefore a random-effects model was employed. The test for subgroup differences was not statistically significant (*P* = 0.535). These results indicate that the severity of disc degeneration as assessed by Pfirrmann grading is not significantly correlated with postoperative recurrence risk. For details, see Figure 12.

#### 3.3.10 Influence of smoking on recurrence

**Figure 13:**
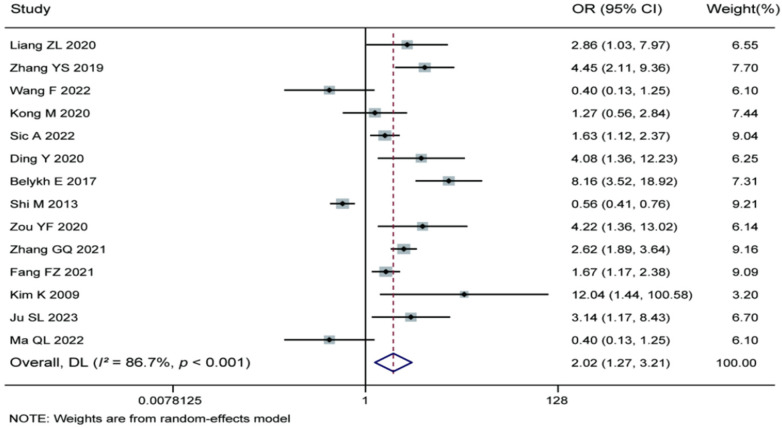
Forest plot of the association between smoking and recurrence.

Meta-analysis of the 14 included studies demonstrated that smoking was a significant risk factor for recurrence after percutaneous endoscopic lumbar discectomy (pooled OR = 2.02, 95% CI: 1.27–3.21). Significant heterogeneity was observed among the studies (*I^2^* = 86.7%, *P* < 0.001), and therefore a random-effects model was applied. The results indicate that smokers have a significantly higher risk of recurrence following percutaneous endoscopic lumbar discectomy compared to non-smokers. Clinically, patients should be advised to quit smoking prior to surgery to reduce the recurrence risk. For details, see Figure 13.

#### 3.3.11 Influence of nucleus pulposus degeneration grade on recurrence

**Figure 14:**
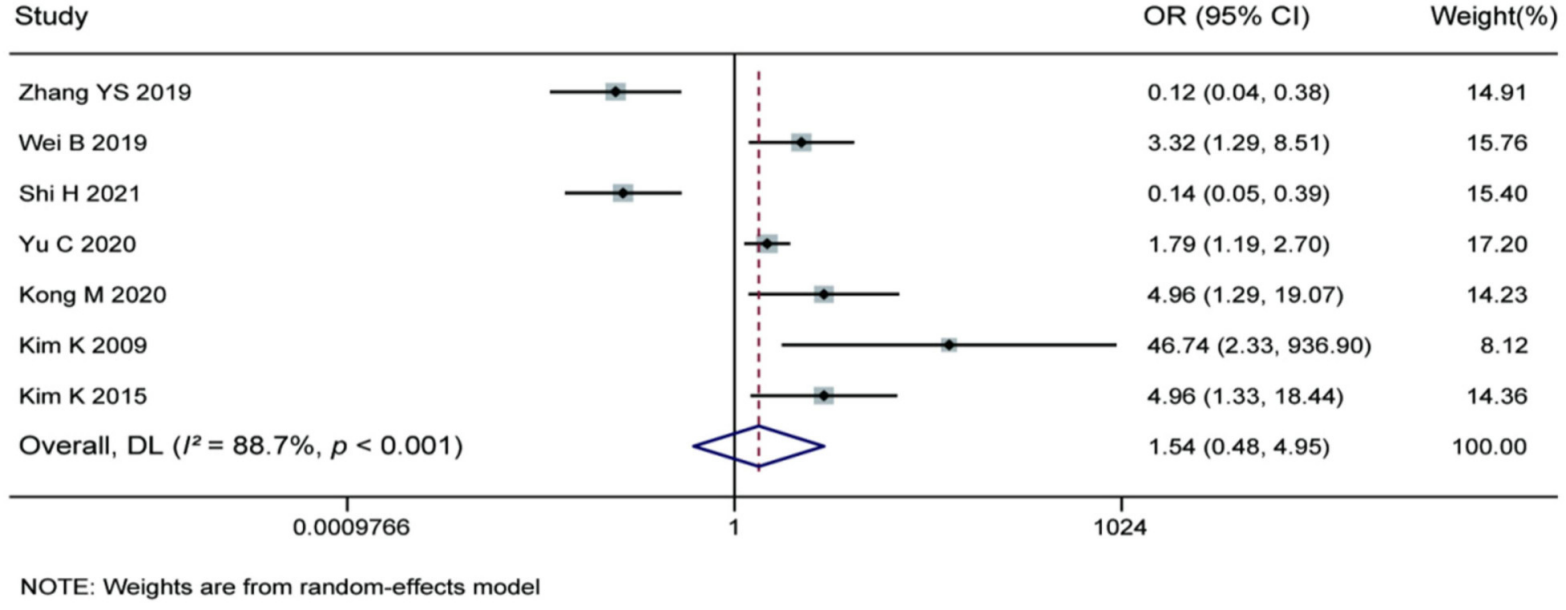
Forest plot of the association between nucleus pulposus degeneration grade and recurrence.

Meta-analysis of the 7 included studies investigating the association between nucleus pulposus degeneration grade and recurrence risk after percutaneous endoscopic lumbar discectomy revealed substantial heterogeneity among the studies (*I^2^* = 88.7%, *P* < 0.001). Although some individual studies suggested a protective effect for higher degeneration grades (OR < 1), others indicated it was a risk factor (OR > 1). The overall pooled effect size calculated using a random-effects model (OR = 1.54) did not reach statistical significance (95% CI: 0.48–4.95). Current evidence does not support nucleus pulposus degeneration grade as an independent influencing factor for postoperative recurrence. The inconsistency in results suggests the potential presence of effect modifiers or methodological variations, necessitating further high-quality studies with greater homogeneity for validation. For details, see Figure 14.

#### 3.3.12 Influence of intraoperative annulus fibrosus rupture on recurrence

**Figure 15:**
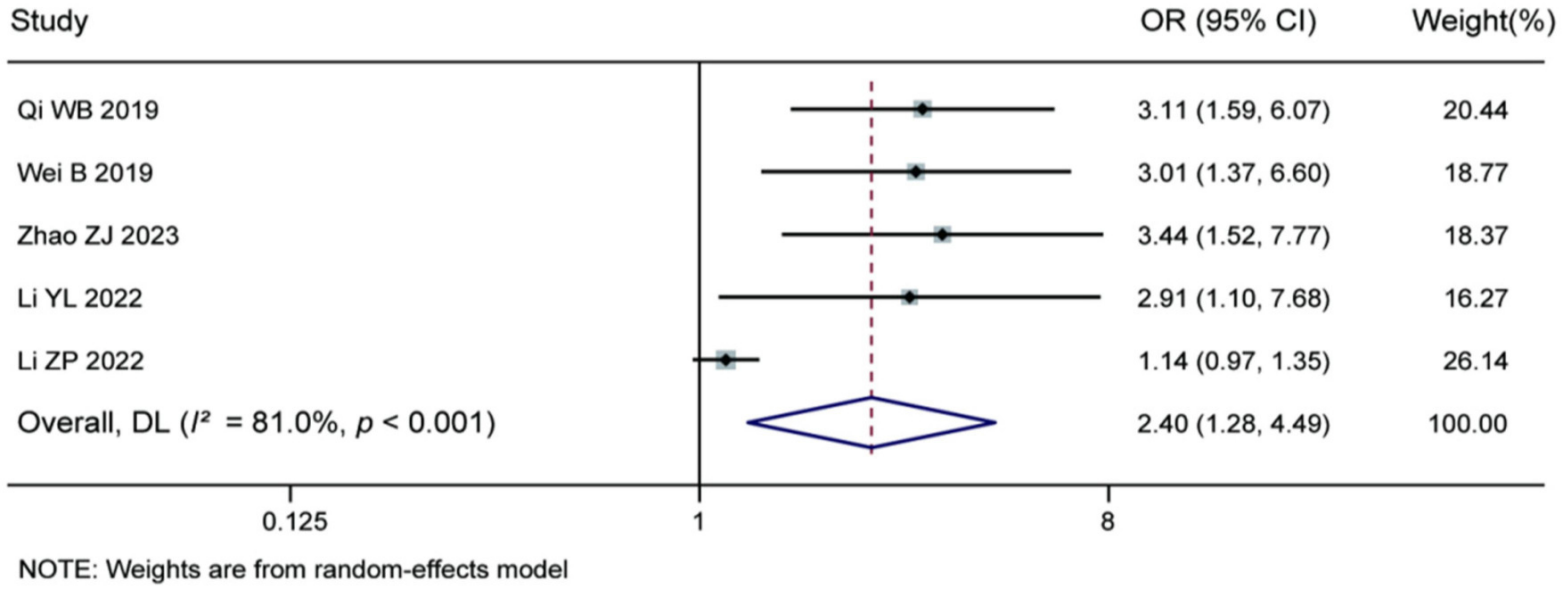
Forest plot of the association between intraoperative annulus fibrosus rupture and recurrence.

Meta-analysis of the 5 included studies demonstrated that intraoperative annulus fibrosus rupture was a significant risk factor for recurrence after percutaneous endoscopic lumbar discectomy (pooled OR = 2.40, 95% CI: 1.28–4.49). Significant heterogeneity was observed among the studies (*I^2^* = 81.0%, *P* < 0.001), and therefore a random-effects model was applied. The results indicate that disruption of the annulus fibrosus integrity during surgery significantly increases the risk of postoperative recurrence. It is recommended that surgeons should endeavor to avoid annulus fibrosus rupture during the procedure. For details, see Figure 15.

#### 3.3.13 Influence of sagittal range of motion on recurrence

**Figure 16:**
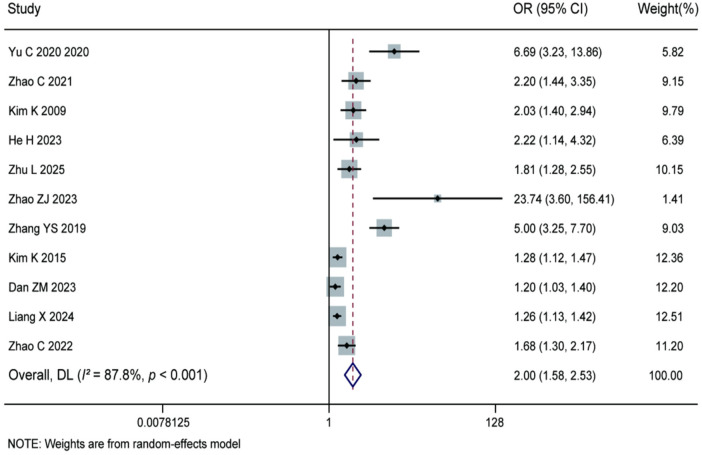
Forest plot of the association between sagittal range of motion and recurrence.

Meta-analysis of the 11 included studies demonstrated that a greater sagittal range of motion was a significant risk factor for recurrence after percutaneous endoscopic lumbar discectomy (pooled OR = 2.00, 95% CI: 1.58–2.53). Significant heterogeneity was observed among the studies (*I^2^* = 87.8%, *P* < 0.001), and therefore a random-effects model was applied. The results indicate that excessive lumbar sagittal range of motion significantly increases the risk of postoperative recurrence. It is recommended that clinical attention should be paid to the preoperative assessment of spinal mobility and that postoperative activity management should be reinforced. For details, see Figure 16.

#### 3.3.14 Publication bias analysis

**Figure 17:**
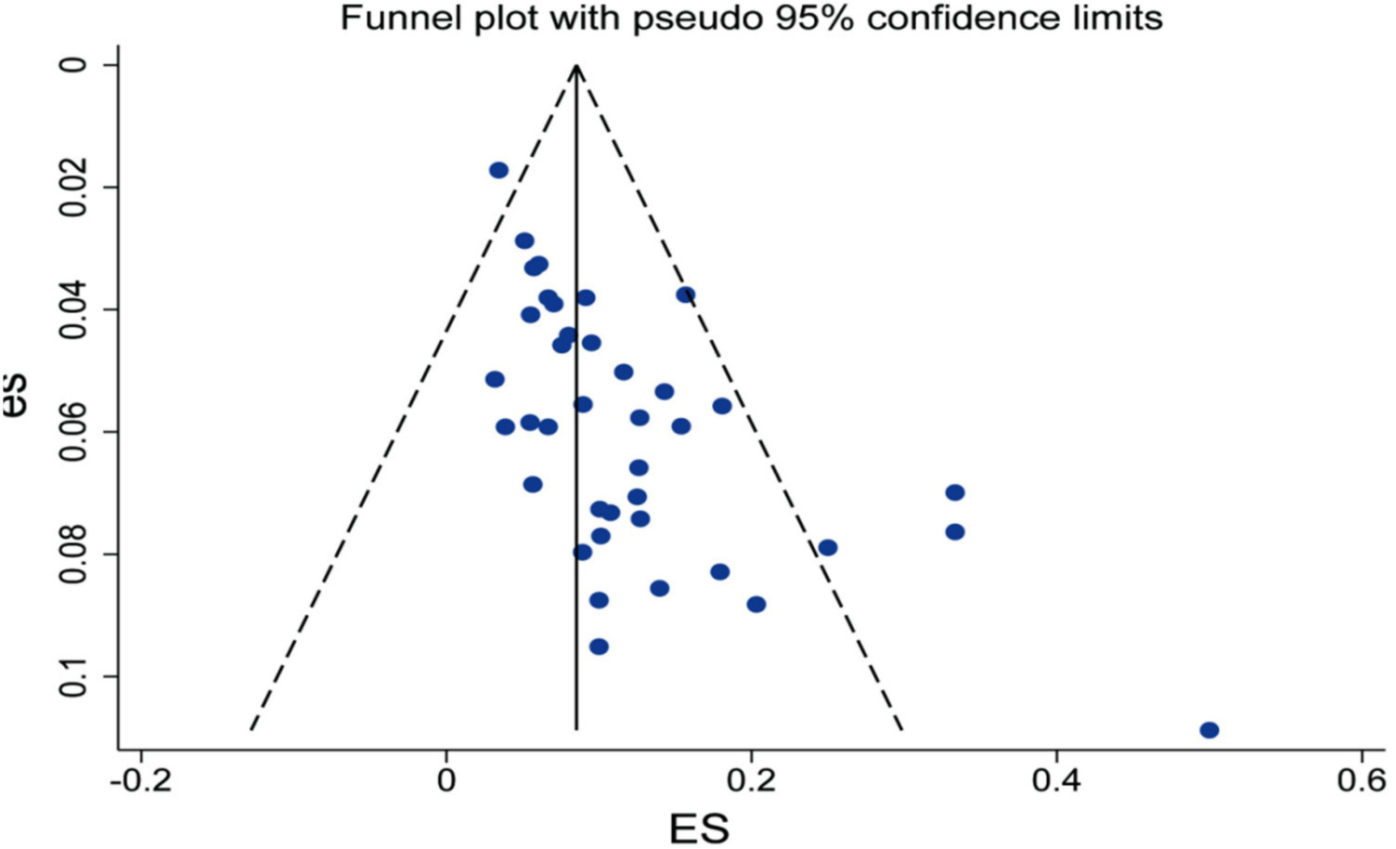
Publication bias funnel plot.

Publication bias in the meta-analysis of risk factors for recurrence after percutaneous endoscopic lumbar discectomy for lumbar disc herniation was assessed using funnel plots. As shown in the figure, the data points were distributed relatively symmetrically and clustered predominantly at the top of the funnel, with most falling within the pseudo 95% confidence limits. This pattern suggests a low likelihood of substantial publication bias. However, minor asymmetry was observed in the lower precision region at the bottom, indicating that potential bias due to the absence of small-scale studies cannot be entirely ruled out. Overall, the results of this meta-analysis are considered reasonably robust, and the impact of publication bias on the pooled effect size is likely to be limited. For details, see Figure 17.

#### 3.3.15 Sensitivity analysis

**Figure 18:**
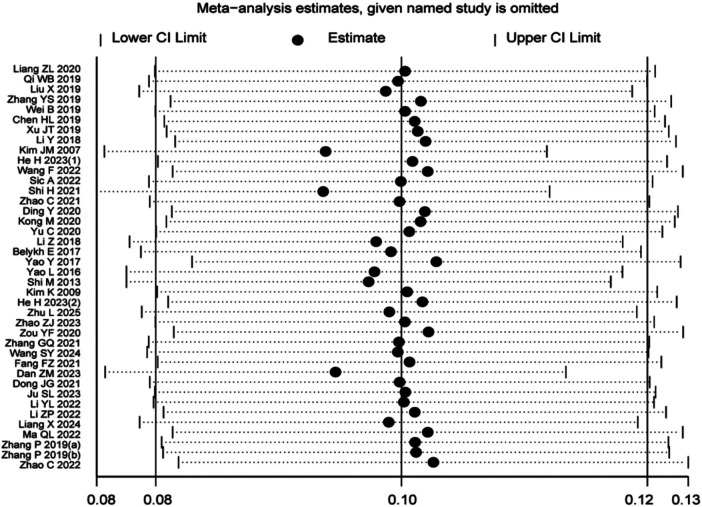
Sensitivity analysis diagram.

The results of the sensitivity analysis showed that after sequentially excluding each individual study, the pooled estimates ranged from 0.08 to 0.13. All recalculated estimates remained within the 95% confidence interval of the overall pooled effect size (0.08–0.13), with no obvious deviations observed. This indicates that the results of the present meta-analysis are robust and were not significantly influenced by any single study, supporting the reliability of the conclusion. For details, see Figure 18.

#### 3.3.16 Heterogeneity assessment

The results presented in Table 4 indicate significant heterogeneity differences among the various risk factors and their subgroups. The analysis of age demonstrated substantial heterogeneity in the overall analysis (*I^2^* = 79.5%, *P* < 0.001), particularly within the ≥60 years subgroup (*I^2^* = 80.1%) and the continuous variable analysis (*I^2^* = 86.5%), whereas the ≥50 years subgroup showed excellent homogeneity (*I^2^* = 0.0%). Analysis of body mass index also revealed substantial overall heterogeneity (*I^2^* = 61.2%), which was more pronounced in the ≥25 kg/m^2^ subgroup (*I^2^* = 70.5%). Regarding imaging factors, all subgroups and the overall analysis of Modic changes exhibited very low heterogeneity (*I^2^* = 0.0%), indicating highly consistent results across studies. The analysis of lumbar disc herniation types also showed good homogeneity (overall *I^2^* = 5.4%). However, it is noteworthy that the test for subgroup differences was statistically significant (*P* = 0.032), suggesting a significant difference in effect sizes between the different classification subgroups. The analysis of postoperative activity intensity indicated acceptable overall heterogeneity (*I^2^* = 46.5%), but moderate heterogeneity was observed within the <5 level subgroup (*I^2^* = 64.8%). Analyses of both Pfirrmann grading and nucleus pulposus degeneration grade showed high heterogeneity (overall *I^2^* = 68.4% and 76.1%, respectively), with heterogeneity being particularly notable in the grade IV Pfirrmann subgroup (*I^2^* = 77.5%). These heterogeneity results suggest that the findings for factors such as age, body mass index, Pfirrmann grading, and nucleus pulposus degeneration grade require cautious interpretation, as their effect sizes may be influenced by differences in study design, population characteristics, or measurement methods. In contrast, the results for factors like Modic changes and lumbar disc herniation types are more stable and reliable. The significant subgroup differences observed for age and lumbar disc herniation types (*P* < 0.05) provide an important basis for risk stratification in clinical practice.

**Table 4:** Heterogeneity analysis.

Indicator	Subgroup	Cochran's Q	df	*P*-value	*I^2^*
Age	≥60 years	20.10	4	< 0.001	80.1%
	≥50 years	2.01	6	0.919	0%
	Continuous variable	52.04	7	< 0.001	86.5%
	Overall	92.89	19	< 0.001	79.5%
	Between subgroups	15.05	2	0.001	/
Body mass index	<25 kg/m^2^	13.28	7	0.065	47.3%
	≥25 kg/m^2^	30.53	9	< 0.001	70.5%
	Overall	43.83	17	< 0.001	61.2%
	Between subgroups	0.60	1	0.437	/
LDH type	Bulging/Protruded	3.25	5	0.662	0%
	Protruded/Sequestrated	0.61	2	0.737	0%
	Overall	8.46	8	0.390	5.4%
	Between subgroups	4.60	1	0.032	/
Modic changes	Grade II	2.57	6	0.860	0%
	Yes&No	2.34	3	0.505	0%
	Overall	5.05	10	0.888	0%
	Between subgroups	0.14	1	0.711	/
Postop activity intensity	≥5 level	0.28	1	0.596	0%
	<5 level	8.52	3	0.036	64.8%
	Overall	9.35	5	0.096	46.5%
	Between subgroups	0.55	1	0.458	/
Pfirrmann grade	Grade III–IV	1.78	1	0.182	43.9%
	Grade IV	13.34	3	0.004	77.5%
	Overall	15.81	5	0.007	68.4%
	Between subgroups	0.38	1	0.535	/
Nucleus Pulposus Degeneration	Grade II–III	1.98	4	0.740	0%
	Grade IV	0.02	1	0.891	0%
	Overall	25.07	6	< 0.001	76.1%
	Between subgroups	23.08	1	< 0.001	/

*I^2^* quantifies the proportion of total variation due to heterogeneity (0–40%: might not be important; 30–60%: moderate; 50–90%: substantial; 75–100%: considerable). A between-subgroups *P*-value < 0.05 indicates a significant difference in effect sizes across subgroups.

## 4 Discussion

### 4.1 Summary of evidence

With the continuing trend of population aging and significant changes in modern lifestyles, the prevalence of LDH has been increasing annually. Epidemiological studies indicate that the overall incidence of LDH is approximately 2%–3%, with rates of about 4.8% in men and 2.5% in women over 35 years of age (47). Against this clinical backdrop, PTED has gradually emerged as an important surgical option for treating LDH, widely accepted by patients due to its significant advantages, including minimal trauma, preservation of bone structures, and rapid postoperative recovery (48). Compared to traditional standard discectomy and fenestration discectomy, PTED demonstrates higher surgical success rates and lower complication rates (49, 50). Furthermore, compared to microendoscopic discectomy techniques, PTED shows superior minimally invasive advantages in further reducing tissue damage, decreasing intraoperative blood loss, and shortening postoperative recovery time (51). However, this procedure still faces the non-negligible issue of postoperative recurrence. Literature reports indicate that the postoperative recurrence rate for LDH ranges between 7.5% and 18.1%, with complex and varied causative factors that have not reached a consensus (52, 53). Current understanding suggests numerous factors influencing recurrence after PTED, potentially involving aspects such as the patient's own disease progression, surgery-related factors, and non-surgical conditions. Recurrence often results from the interplay of multiple mechanisms. Significant heterogeneity exists in domestic and international studies regarding patient baseline characteristics, surgical indications, methodological approaches, surgeons' technical skills, follow-up duration, and sample sizes. This heterogeneity leads to inconsistencies and controversies in identifying risk factors for recurrence, highlighting an urgent need for further clarification through more high-quality, large-sample clinical research.

#### 4.1.1 Patient demographic and baseline characteristic factors

This study identified that advanced age (pooled OR = 1.21), higher BMI (pooled OR = 1.30), smoking (pooled OR = 2.02), and diabetes mellitus (pooled OR = 2.34) were all independent risk factors for recurrence after PELD. Age is a key factor influencing disc degeneration and repair capacity. The subgroup analysis in this study showed a significantly increased recurrence risk in the ≥60 years age group (OR = 2.50). In the study by Kim et al. (16), which included a considerable proportion of elderly patients, the recurrence rate was as high as 50% in patients with a mean age of 47.4 years. This high risk is attributed to the combined effect of decreased tissue healing capacity and exacerbated disc degeneration in older adults. Correspondingly, Zhang et al. (11), in their analysis of 285 patients, found that for every 10-year increase in age, the recurrence risk increased approximately 1.3-fold. The underlying mechanism may involve an age-related decrease in proteoglycan content within the nucleus pulposus, reduced toughness of the annulus fibrosus, leading to poorer postoperative mechanical stability of the disc, and a significant decline in tissue self-repair ability.

Higher BMI (≥25 kg/m^2^) is another important modifiable risk factor. This study indicated a 30% increased recurrence risk in this population. An elevated BMI increases the mechanical load on the lumbar spine and intervertebral discs, raising the risk of postoperative recurrence. Obesity is often associated with chronic inflammation and diabetes. Substances such as tumor necrosis factor-alpha (TNF-α), interleukin-1β (IL-1β), and advanced glycation end products can accelerate disc degeneration, contributing to recurrent LDH (54). For instance, in a study of 130 patients by Wei et al. (12), the recurrence rate was significantly higher in patients with a BMI >28 kg/m^2^ (15.4%) compared to the normal BMI group (6.1%). The pathophysiological mechanisms are multifaceted. Firstly, the increased mechanical load directly elevates pressure on the lumbar discs, particularly at weight-bearing segments like L4-L5 and L5-S1. Secondly, obesity is often accompanied by a chronic, low-grade systemic inflammatory state; inflammatory cytokines (e.g., IL-6, TNF-α) secreted by adipocytes can accelerate the degradation of the disc matrix and hinder postoperative repair. Furthermore, obesity often increases the technical difficulty of the surgical procedure, such as challenges in puncture localization and suboptimal placement of the working channel, which may indirectly affect the surgical outcome (55, 56).

Smoking is one of the strongest behavioral risk factors for postoperative recurrence (OR = 2.02). Among the 14 studies included, a large-sample study (*n* = 690) by He et al. (30) indicated that the recurrence risk in smokers was 2.5 times that of non-smokers. Harmful substances like nicotine and carbon monoxide can cause vasospasm and microcirculatory impairment around the disc, leading to reduced nutrient supply to the nucleus pulposus and accumulation of metabolic waste. Concurrently, smoking inhibits fibroblast activity, delaying the healing of tears in the annulus fibrosus, thereby creating a pathway for new herniation. In clinical practice, preoperative smoking cessation should be emphasized as mandatory patient education, and the duration of cessation might be considered a factor in determining the optimal timing for surgery (57).

Diabetes mellitus, with its significant metabolic disturbances, increases the recurrence risk by 2.34 times. Animal experiments by Zheng Xuhao et al. (58) suggested that diabetes might lead to decreased nutrient supply and impaired metabolite exchange in the intervertebral disc, accelerating disc degeneration, although its direct relationship with postoperative recurrence remained unclear. This study, however, demonstrates that a history of diabetes is an influencing factor for postoperative recurrence. For example, the study by Ding et al. (22) found a significantly higher recurrence rate in patients with diabetes (12.1%) compared to non-diabetic patients (4.9%). Long-term hyperglycemia damages disc health through multiple pathways: firstly, the accumulation of advanced glycation end products (AGEs) increases collagen cross-linking and reduces elasticity, making the annulus fibrosus more fragile; secondly, diabetic microangiopathy reduces endplate permeability, further exacerbating impaired nutrient supply to the disc; thirdly, the hyperglycemic environment predisposes individuals to low-grade infections, which could be a hidden factor in recurrence. For diabetic patients, strict perioperative blood glucose control may be a key measure to reduce recurrence risk (59, 60).

#### 4.1.2 Imaging and pathological classification factors

Preoperative imaging assessment holds significant value for predicting recurrence risk. This study focused on factors such as Modic changes, type of lumbar disc herniation, and Pfirrmann grade. Modic changes, particularly type II (fatty degeneration), were significantly associated with an increased recurrence risk (OR = 1.87). Primarily assessed via MRI, Modic changes manifest as alterations in the signal intensity of the vertebral endplates and adjacent bone marrow, and are closely associated with LDH, disc degeneration, and low back pain (61, 62). For instance, in the study by Zhang et al. (34), the recurrence rate in patients with Modic type II changes was as high as 18.3%, far exceeding the rate in the group without Modic changes (6.1%). Modic type II changes typically indicate marrow fat replacement and chronic inflammation. This environment may accelerate the degeneration and re-herniation of residual nucleus pulposus through the release of inflammatory factors (e.g., IL-1β, TNF-α) and proteases. Furthermore, Modic changes are often accompanied by endplate dysfunction, which impairs nutrient diffusion to the disc, hindering postoperative repair. Therefore, for patients presenting with Modic changes, especially type II, on preoperative MRI, the relatively higher recurrence risk should be communicated, and considerations for more thorough decompression or stricter postoperative follow-up may be warranted (63, 64).

The type of lumbar disc herniation is a strong predictor of recurrence. This study found that sequestrated disc herniation carried the highest recurrence risk (OR = 3.75), followed by protruded herniation, while bulging herniation had the lowest risk. This aligns with the findings of Chen et al. (13), who reported a 3.2 times higher recurrence rate for sequestrated fragments compared to contained herniations in their study of 212 patients. The mechanism primarily lies in the fact that sequestrated herniations often involve larger tears in the annulus fibrosus that are less likely to heal spontaneously, leaving a larger residual cavity post-discectomy that is more susceptible to re-herniation under spinal load (53). Additionally, sequestrated fragments are often tightly adherent to the dural sac or nerve roots; to avoid neural injury, surgeons might not be able to completely remove the compressive material, potentially leaving remnants that serve as a nidus for recurrence. For sequestrated herniations, surgeons should consider using a larger working channel or combined techniques to ensure clear visualization for thorough fragment removal and evaluate the potential application of annuloplasty (65).

Notably, Pfirrmann grade did not show a statistically significant association with recurrence in this study (pooled OR = 1.28, 95% CI: 0.95–1.60). This result differs from conclusions drawn in some previous studies. Potential reasons for this inconsistency include: Firstly, the Pfirrmann grading system is a subjective morphological classification based on T2-weighted images, susceptible to inter-observer and even intra-observer variability, which is reflected in the high heterogeneity observed for this factor (I^2^ = 68.4%) in our analysis. Secondly, intervertebral disc degeneration is a multi-dimensional process, and assessing it solely based on signal intensity and water content (the core of Pfirrmann grading) might be insufficient to fully capture its mechanical stability and biological activity (66, 67). Future research may need to incorporate more objective, quantitative imaging biomarkers, such as T2-mapping (assessing matrix content), dGEMRIC (assessing glycosaminoglycan content), or DTI (assessing annulus fibrosus structural integrity), to more accurately evaluate the relationship between disc degeneration and recurrence risk.

#### 4.1.3 Surgical technique and intraoperative factors

Among surgeon-controllable factors, intraoperative annulus fibrosus rupture is particularly noteworthy, as it increases the recurrence risk by 2.40 times. The integrity of the annulus fibrosus serves as a critical barrier maintaining the intrinsic biomechanical stability of the intervertebral disc. Once ruptured, especially with a tear oriented towards the spinal canal, it loses its restraining force on the nucleus pulposus tissue. Multiple studies concur on its close association with postoperative recurrence (16, 68–70), a view consistent with the results of this analysis.However, the extent of nucleus pulposus removal presents a paradoxical dynamic. While more extensive removal leaves less residual material, potentially lowering recurrence, excessive removal can accelerate degeneration of the remaining disc tissue, increasing the risk of adjacent segment pathology. This necessitates that clinicians tailor the extent of decompression based on individual patient circumstances. In the study by Ding et al. (22), the postoperative recurrence rate was as high as 21.4% in patients where annular rupture was noted intraoperatively, compared to only 5.1% in the non-rupture group. The occurrence of annular rupture may be related to the following factors: 1) The surgeon pursuing overly aggressive decompression by excessively removing nucleus pulposus, including from normal areas; 2) Improper manipulation of instruments (e.g., pituitary rongeurs, radiofrequency electrodes) causing iatrogenic damage to the inner layers of the annulus; 3) Inherent fragility and degeneration of the patient's annulus, making it susceptible to tearing even with minimal manipulation (71). Therefore, surgeons should adhere to the philosophy of “targeted decompression,” aiming to relieve neural compression rather than striving for a radiologically “complete” removal of disc material. Meticulous intraoperative technique is crucial to avoid instruments being advanced too deeply into the disc space and causing excessive traction or tearing. In recent years, the development of annulus fibrosus repair techniques offers a potential solution to this challenge. Preliminary studies suggest that using annular closure devices to seal the annular defect can significantly reduce early postoperative recurrence rates, although their long-term efficacy and cost-effectiveness require further high-level evidence for support.

#### 4.1.4 Surgical approach selection and the influence of disc herniation type

This study confirmed that the type of lumbar disc herniation (e.g., sequestrated) is a significant risk factor for recurrence. However, the recurrence risk may not only be related to the morphology of the herniation itself but also to whether the chosen surgical approach allows for optimal decompression of that specific type. Percutaneous Endoscopic Lumbar Discectomy primarily includes two main approaches: PTED and PIED, each with its own emphasis in indications. For lateral or extraforaminal lumbar disc herniations, the PTED is widely considered one of the optimal choices. This approach can provide an ideal working channel directly to the target herniation, offering a direct field of view that allows for nucleus pulposus removal under direct vision. It avoids extensive facet joint resection often required in traditional open surgery, potentially enabling more complete decompression and reducing the risk of recurrence due to residual fragments (72). Conversely, for central or massive paracentral herniations, PTED alone may face technical challenges due to the working angle, sometimes making it difficult to safely address contralateral or ventral fragments. In such cases, the PIED or biportal endoscopic techniques may offer a superior visual field and operational space (73). It is noteworthy that while these alternative endoscopic techniques provide solutions, their long-term efficacy superiority over classical microdiscectomy still requires further high-level evidence from prospective comparative studies. Most studies included in this meta-analysis did not further stratify recurrence rates by surgical approach based on the specific location of the herniation (e.g., extraforaminal vs. central). This could be a potential source of heterogeneity in some of our results. Future research should report in more detail the matching between surgical approach and herniation type to allow for a more precise assessment of recurrence risk for each technique in specific patient subgroups.

#### 4.1.5 Postoperative rehabilitation and biomechanical factors

Postoperative management is equally critical. This study found that both engaging in high-intensity activities too soon after surgery (pooled OR = 1.83) and having a greater preoperative lumbar sagittal range of motion (pooled OR = 2.00) significantly increased the risk of recurrence. The biomechanical environment of the lumbar spine is particularly vulnerable in the early postoperative period. Following nucleus pulposus removal, the intradiscal pressure decreases, and the annulus fibrosus tear is in the process of healing. If subjected to excessive load during this phase, especially combined stresses from flexion, rotation, and axial compression, there is a high risk of recurrent herniation through the unhealed tear. Shimi et al. (28) reported that high-intensity and heavy physical work postoperatively significantly predisposes patients to recurrent lumbar disc herniation. This study confirms a close association between postoperative strenuous physical work and recurrence after PELD. The study by Wei et al. (12) clearly reported that patients engaged in heavy physical labor (e.g., lifting, bending tasks) within 3 months after surgery had a 2.5 times higher recurrence risk compared to those performing light activities. Management of postoperative activity intensity should be individualized. For young patients without Modic changes or annular rupture, encouraging early non-loading or low-load activities (such as walking) can promote blood circulation and tissue healing. However, for high-risk patients who are older, obese, or had intraoperative annular rupture, prolonged use of a lumbar brace and strict avoidance of high-intensity activities for at least 3 to 6 months is necessary. Core muscle stability training under the guidance of a physical therapist is particularly important. Strengthening deep core muscles like the transversus abdominis and multifidus can share the load on the lumbar spine, forming an “internal biomechanical brace” that creates a more stable environment for disc healing (74, 75). An increased sagittal range of motion implies higher lumbar flexibility but may also indicate segmental instability. Segments with greater mobility subject the annulus fibrosus to higher shear and tensile forces, posing a greater challenge to postoperative healing (76). The study by Yu et al. (24) found that the average preoperative lumbar flexion-extension range of motion was significantly higher in the recurrence group (45.6°) compared to the non-recurrence group (32.1°). Analyses by Wei Bing (12) and Kim et al. (54) on postoperative intervertebral mobility found that greater mobility was associated with a higher likelihood of recurrence. For such patients, beyond general activity restrictions, postoperative rehabilitation should focus more on motor control training rather than flexibility training. Patients should be taught how to maintain a neutral spine and coordinate movements during daily activities (e.g., bending to pick up objects, rising from a chair), avoiding end-range flexion and extension movements.

### 4.2 Limitations of the article

This systematic review and meta-analysis has several limitations that should be considered: ① The included original studies were all observational (case-control or retrospective cohort) in design, which carry inherent limitations in participant selection, measurement of exposure factors, and particularly in the control for important confounding factors (with a compliance rate of only 38.46% for this item). These limitations may have introduced selection bias and confounding bias. ② The meta-analysis results indicated moderate to high heterogeneity for most influencing factors. The sources of this heterogeneity include differences across studies in population characteristics, definitions and measurement methods of predictors, surgical technique details, and follow-up durations, which limit the precision of the pooled effect estimates. ③ The literature search was restricted to Chinese and English databases and did not include grey literature, potentially introducing language bias and publication bias. ④ This study only performed univariate pooled analyses and could not delve into the complex interactions among multiple factors. These limitations may affect the robustness and generalizability of the findings to some extent, and conclusions should be interpreted with caution.

### 4.3 Conclusion

This meta-analysis demonstrates that recurrence following PELD is a clinical outcome influenced by multiple factors. Advanced age, higher body mass index, smoking, diabetes mellitus, Modic changes (especially type II), sequestrated disc herniation, intraoperative annulus fibrosus rupture, early postoperative high-intensity activity, and increased lumbar sagittal range of motion were identified as significant independent risk factors. These findings suggest that a more comprehensive perioperative management strategy should be implemented for patients with these characteristics, including strict adherence to surgical indications, optimized surgical techniques to preserve annulus fibrosus integrity, and the development of individualized, stepwise rehabilitation plans. Future research should focus on integrating these core predictors to develop and externally validate efficient clinical risk prediction models, thereby providing an evidence-based foundation for the precise prevention and management of recurrence after PELD.

## Data Availability

The original contributions presented in the study are included in the article/Supplementary Material, further inquiries can be directed to the corresponding author.
